# Mutant p53 gain of function underlies high expression levels of colorectal cancer stem cells markers

**DOI:** 10.1038/s41388-017-0060-8

**Published:** 2018-01-18

**Authors:** Hilla Solomon, Nathan Dinowitz, loannis S. Pateras, Tomer Cooks, Yoav Shetzer, Alina Molchadsky, Meital Charni, Stav Rabani, Gabriela Koifman, Ohad Tarcic, Ziv Porat, Ira Kogan-Sakin, Naomi Goldfinger, Moshe Oren, Curtis C. Harris, Vassilis G. Gorgoulis, Varda Rotter

**Affiliations:** 1 Department of Molecular Cell Biology, Weizmann Institute of Science, Rehovot, Israel; 2 Molecular carcinogenesis Group, Department of Histology-Embryology, School of Medicine, University of Athens, Athens, Greece; 3 Laboratory of Human Carcinogenesis, National Cancer Institute, National Institute of Health, Bethesda, MD, USA; 4 The Flow Cytometry Unit, Life Sciences Faculty, Weizmann Institute of Science, Rehovot, Israel

## Abstract

Emerging notion in carcinogenesis ascribes tumor initiation and aggressiveness to cancer stem cells (CSCs). Specifically, colorectal cancer (CRC) development was shown to be compatible with CSCs hypothesis. Mutations in p53 are highly frequent in CRC, and are known to facilitate tumor development and aggressiveness. Yet, the fink between mutant p53 and colorectal CSCs is not well-established. In the present study, we set to examine whether oncogenic mutant p53 proteins may augment colorectal CSCs phenotype. By genetic manipulation of mutant p53 in several cellular systems, we demonstrated that mutant p53 enhances colorectal tumorigenesis. Moreover, mutant p53-expressing cell lines harbor larger sub-populationss of cells highly expressing the known colorectal CSCs markers: CD44, Lgr5, and ALDH. This elevated expression is mediated by mutant p53 binding to CD44, Lgr5, and ALDH1A1 promoter sequences. Furthermore, ALDH1 was found to be involved in mutant p53-dependent chemotherapy resistance. Finally, analysis of ALDH1 and CD44 in human CRC biopsies indicated a positive correlation between their expression and the presence of oncogenic p53 missense mutations. These findings suggest novel insights pertaining the mechanism by which mutant p53 enhances CRC development, which involves the expansion of CSCs sub-populations within CRC tumors, and underscore the importance of targeting these sub-populations for CRC therapy.

## Introduction

Colorectal cancer (CRC) is the third most frequent cause for cancer-related deaths in the world [1], Its development is associated with series of defined genetic alterations that promote the transformation of normal epithelial mucosa into carcinoma, including aberrations in APC, K-Ras, and p53 [2, 3]. However, recent studies revealed inter-tumoral as well as intra-tumoral heterogeneity, associated with changes in gene expression or in epigenetics [1, 4]. This heterogeneity can be explained by the hierarchical model for cancer development, which predicts that only a small subset of cells within tumors, termed cancer stem cells (CSCs), has the ability to proliferate and propagate the tumor as well as to differentiate into various lineages [5]. Moreover, it is accepted that CSCs are the entity that endows tumors with chemotherapy resistance, and are responsible for tumor relapse [1, 6].

The epithelial homeostasis of the intestine relies on the presence of highly active normal stem cells in the bottom of the intestine crypt that self-renew, while generating new functional epithelia in high frequency [7]. However, when normal stem cells gain genetic or epigenetic modifications they can evolve into CSCs, leading to cancer development [6, 8]. Thus, to maintain normal homeostasis, stem cells of the intestine system must be tightly regulated.

The tumor-suppressor p53 was found to ensure the quality and genomic stability of stem cells; hence, it serves as barrier to CSCs formation [6]. Its intact functionality is crucial for the maintenance of healthy cells and tissues, thus it is not surprising that p53 is the most frequently mutated gene in human cancer [9]. When mutated, p53 does not only lose its tumor-suppressive functions, rather it gains additional oncogenic functions, a phenomenon termed mutant p53 gain of function (GOF). Ample experimental evidence suggest that mutant p53 GOF mediates oncogenic properties such as sustained proliferation, cell death resistance, invasion and metastasis, and tumor-promoting inflammation [10–12].

p53 was found to be mutated in about 40 percent of CRC cases. The most frequently mutated codons in p53 are 175, 248, and 273 (IARC TP53 Database R18, April 2016) [13]. Interestingly, these missense mutations belong to two p53 mutations sub-groups that define p53 mutation type according to their impact on the DBD folding; “DNA-contact mutations” (R248, R273), and the “p53 conformational mutations” (R175) [14], Indeed, it is well-accepted that mutant p53 plays an important role in CRC development [3]. Accordingly, we previously found that mutant p53 promotes inflammation-associated colorectal cancer [15].

Accumulated data suggest that mutant p53 facilitates the acquisition of CSCs phenotype. This can be deduced by the correlation between mutant p53 and undifferentiated tumors [16] as well as by the malignant phenotype of induced pluripotent stem cells (iPSCs) generated upon reprogramming of mutant p53-expressing mouse embryonic fibroblasts (MEFs) [17]. Interestingly, CSCs properties such as drug resistance and enhanced metastasis seem to interweave with mutant p53 GOF activities [11, 18]. In all, we hypothesized that mutant p53 promotes colorectal tumorigenesis by expanding colorectal CSCs sub-populations.

Here, we manipulated mutant p53 expression in tumor-derived colorectal cell lines and examined its effect on CSCs sub-populations and on tumor aggressiveness. As expected, we found that mutant p53 promotes the tumorigenic potential of colorectal cells as well as confers them with chug resistance. Then, to study the effect of mutant p53 on colorectal CSCs, we examined the expression of three well-established colorectal CSCs markers, Lgr5, ALDH, and CD44 [19] in colorectal cell lines as well as in intestinal organoid, representing a more physiological system. We found that mutant p53-expressing cells harbor larger CD44^Br^, Lgr5^Br^ as well as activated ALDH (ALDH^Br^) sub-populationss compared with p53-deficient cells. Our data suggest that ALDH^Br^ sub-population within mutant p53-expressing cells exhibit self-renewal capacity, and that the chemotherapy resistance that is induced by mutant p53 is mediated by ALDH. Moreover, we demonstrate that mutant p53 induces the expression of CD44, Lgr5, and ALDH by binding to their promoters. Finally, these data were corroborated in human colorectal tumors, in which we found positive correlation between the presence of mutant p53 and ALDH1 as well as CD44 expression.

Our data suggest that the enhanced CRC aggressiveness conferred by mutant p53 is mediated by augmented CSCs phenotype.

## Results

### Mutant p53 gain of function endows colorectal cancer cell lines with higher oncogenic potential

**Fig. 1:**
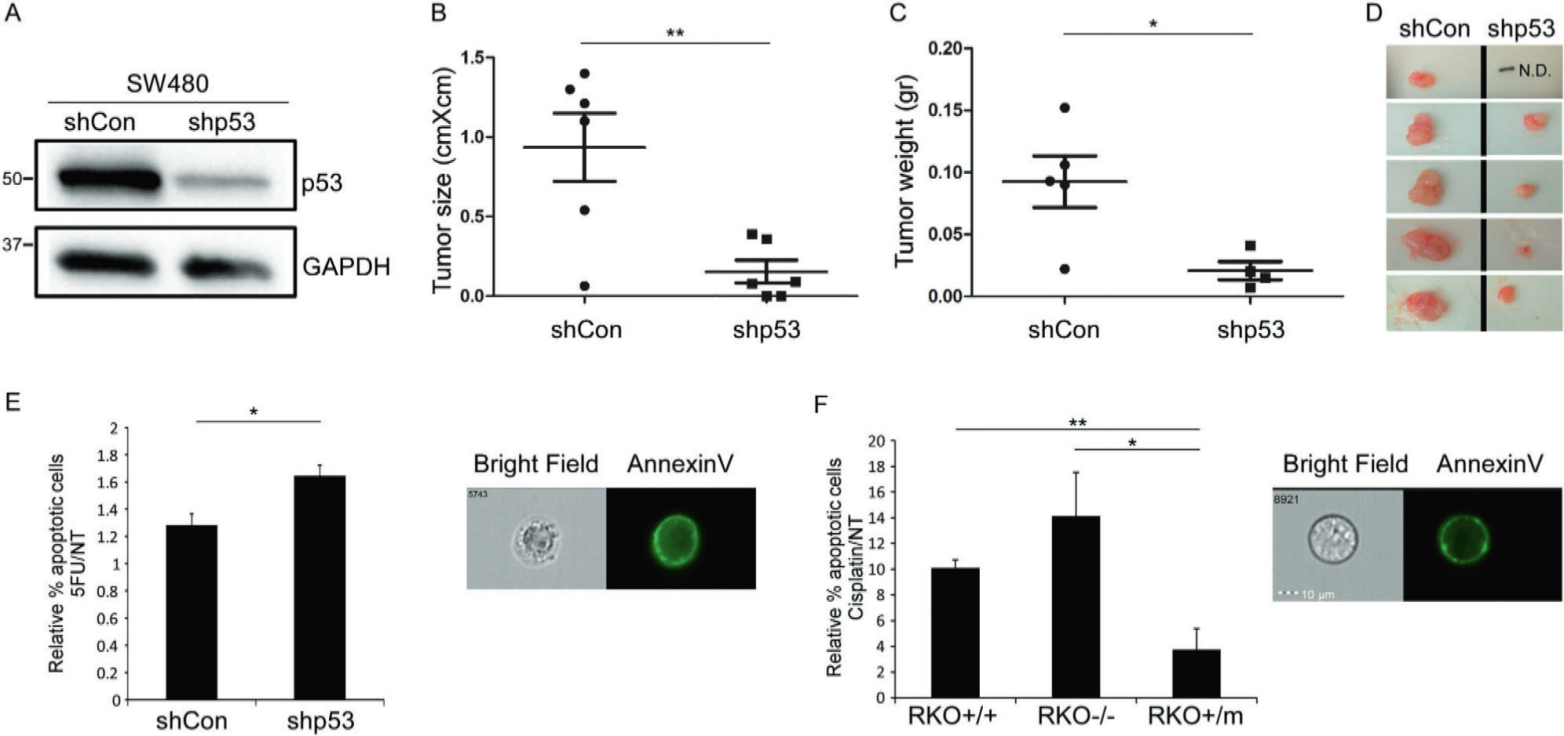
Mutant p53 gain of function endows colorectal cancer cell lines with higher oncogenic potential. The colorectal adenocarcinoma-derived cell line, SW480 that endogenously expresses mutant p53^R273H,P309S^ was stably infected with shRNA against p53 (shp53) or with shRNA against nonspecific sequence (shCon), as a control, **a** Western Blot displays mutant p53 protein levels. GAPDH serves as loading control. This figure combines two independent detections of p53 and GAPDH of the same gel. The two original images are presented in Supplementary Figure 1. **b** The shCon and shp53 SW480 cell lines (2 × 10^5^ or 1 × 10^4^) were injected into left and right limb of nude mice, respectively, followed by tumor growth surveillance. After 8–10 weeks mice were sacrificed and tumors size was measured. Asterisks denotes statistical significance (*p* < 0.01, *n* = 6). **c** Three independent newly infected shCon and shp53 SW480 cell lines were mixed and were injected (7 × 10^5^) into left and right limb of nude mice, respectively, followed by tumor growth surveillance. After 5 weeks mice were sacrificed and tumors weight was measured. Asterisks denotes statistical significance *(p* < 0.05, *n* = 5). **d** Photos of tumors presented in c that illustrate the significant differences in the size of tumors obtained from injection of shCon and shp53 SW480 cells, **e** The established SW480 cell lines were treated with 5-FU (50 μΜ) for 72 h, followed by AnnexinV staining and ImageStream X analysis (Materials and methods). Graph in left panel indicates on percentage of apoptotic cells upon 5-FU treatment, normalized to NT cells. Right panel is a representative photo of an apoptotic cell, **f** RKO isogenic cell lines that express either WTp53 (RKO+/+) or mutant p53^R248W^ (RKO+/m), or knocked-out for p53 (RKO−/−) [20] were treated with cisplatin (2.5 μg/ml) for 72 h, followed by AnnexinV staining and ImageStream X analysis. Graph in left panel indicates on percentage of apoptotic cells upon cisplatin treatment, normalized to NT cells. Right panel is a representative photo of an apoptotic cell. Graphs represent an average of three experiments. Error bars represent SE. Asterisk denotes statistical significance. For more representative photos obtained by ImageStream X see Supplementary Figure 13 A–B

It is well-accepted that mutant p53 possesses GOF activities that confer cells with oncogenic potential [10]. Here, we aimed to examine the tumorigenic potential of mutant p53 in colorectal tumor-derived cell lines. To this end, the colorectal adenocarcinoma-derived cell line, SW480, endogenously expressing mutant p53^R273H,P309S^, was stably introduced with either shRNA against p53 (shp53) to knock-down mutant p53^R273H,P309S^ expression, or as a controls with shRNA against nonspecific sequence (shCon) (Fig. 1a; Supplementary Figure 1, Supplementary Figure 2A) as well as shRNA control against lacZ gene (sh-lacZ) (Supplementary Figure 2A). As a first step, we aimed to evaluate whether mutant p53 confers SW480 cells with tumorigenic potential in vivo. The established SW480 cell lines were injected subcutaneously into immune-compromised mice, followed by tumor growth surveillance. Strikingly, shCon and sh-lacZ cell lines induced detectable tumors, while shp53 cell line gave rise to significantly smaller or undetectable tumors (Fig. 1b–d; Supplementary Figure 2). These results indicate that reduction in mutant p53^R273H,P309S^ protein levels attenuates the tumorigenic potential of the cells and further supports the notion that mutant p53 expression facilitates tumorigenesis in vivo.

An additional oncogenic characteristic mediated by mutant p53 GOF is drug resistance [10]. To challenge this issue we have exposed the shCon and shp53 SW480 cell lines to chemotherapy and assessed their resistance to apoptosis. SW480 cells were treated with the chemotherapy agent, 5-FU, followed by AnnexinV staining. The percentage of apoptotic cells was assessed by ImageStream X that combines fluorescence-activated cell sorting (FACS) with microscopy. This instrument allows visualizing all analyzed cells and assesses staining statistics (Fig. 1). The results obtained show that knockdown of mutant p53^R273H,P309S^ (shp53) significantly increased apoptosis following chemotherapy treatment compared with that seen in the shCon cells, suggesting that mutant p53^R273H,p309S^ protects cells from chemotherapy-induced apoptosis. To corroborate these results in additional colorectal cellular system, we examined the sensitivity of the RKO isogenic cell lines to chemotherapy. RKO cells that endogenously express wild-type p53 (RKO+/+), and their counterparts that were either introduced with mutant p53^R248W^ (RKO+/m), or knocked-out for p53 (RKO−/−) [20] were treated with the chemotherapy agent cisplatin, followed by AnnexinV staining and analysis of apoptosis by ImageStream X (Fig. 1f). The results indicate that introduction of mutant p53^R248W^ to RKO cells significantly reduced apoptosis following chemotherapy treatment compared with both RKO+/+ and RKO−/− cells. This findings support the results obtained from SW480 experiments, thus overall suggesting that mutant p53 protects cells from chemotherapy-induced apoptosis in a GOF manner. To further validate that mutant p53 GOF endows cells with drug resistance we stably introduced mutant p53^R175H^ to HI299 cells that are null for p53 (Supplementary Figure 3) and evaluated their sensitivity to chemotherapy. The established H1299 cells were treated with cisplatin followed by detection of poly (ADP-ribose) polymerase (PARP-1) cleavage that is important for apoptosis [21] (Supplementary Figure 3). In accordance with the colorectal cell lines, mutant p53^R175H^-expressing H1299 cells exhibited reduced levels of PARP-1 cleavage compared with the null cells (Supplementary figure 3). Altogether, these data further support the conclusion that mutant p53 enhances the oncogenic characteristics of cancer cell fines in a GOF manner.

### Mutant p53-expressing colorectal cancer cell lines possess augmented CSCs sub-populations

**Fig. 2:**
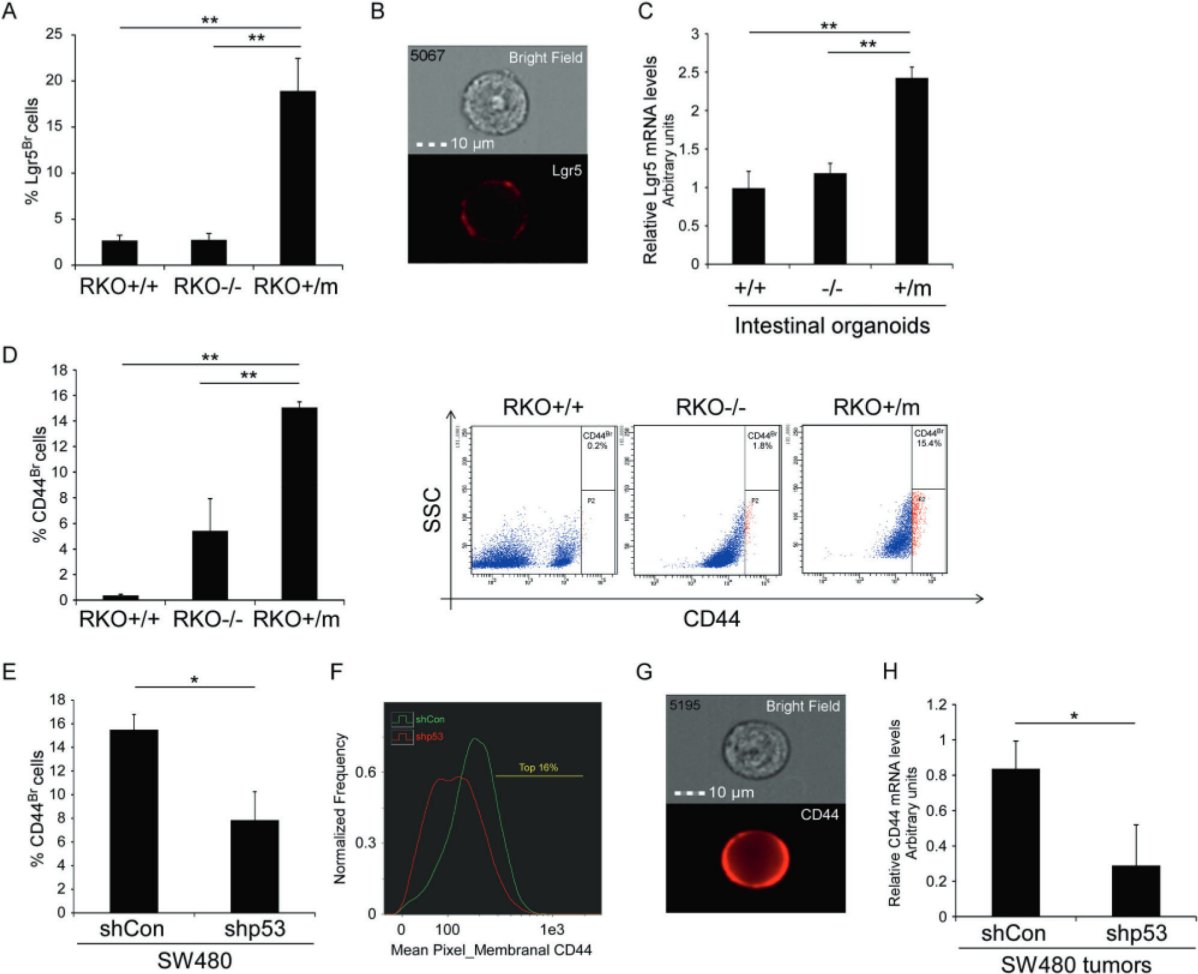
Mutant p53-expressing cells contain larger CD44^Br^ and Lgr5^Br^ sub-populations **a, b** RKO isogenic cell lines were immuno-stained with anti-Lgr5 antibody and the size of Lgr5^Br^ sub-population was measured by ImageStream X. **a** Graph presenting an averaged percentage of Lgr5^Br^ cells obtained from three independent experiments. Error bars represent SE. **b** Representative photo of Lgr5^Br^ cedi, **c** Organoids were produced from intestinal epithelial cells extracted from WTp53, p53 knock-out (p53 KO), or WTp53/mutant p53^R172H^ heterozygous mice, as previously described [15]. Lgr5 mRNA expression levels were measured by qRT-FCR using specific primers. Graph represents an average of three independent organoids pools. Error bars represent SE. **d** RKO isogenic cell lines were immuno-stained with either anti-CD44 antibody that recognizes all CD44 isoforms and the size of CD44^Br^ sub-populations was measured by FACS. Left: Graph presenting an averaged percentage of CD44^Br^ cells obtained from three experiments. Error bars represent SE. Right panel shows representative dot plots indicating on percentage of CD44^Br^ sub-populations, **e-g** The established shCon and shp53 SW480 cells were immuno-stained with either anti-CD44 antibody that recognizes all CD44 isoforms and the size of CD44^Br^ sub-populations was measured by ImageStream X. **e** Graph presenting an averaged percentage of cells obtained from four experiments, **f** Representative plot of the mean pixel intensity of membranal CD44 in shCon and shp53 cell populations, **g** Representative photo of CD44^Br^ cell obtained by ImageStream X. For more representative photos obtained by Image-Stream X see Supplementary Figure 13 C–D. **h** mRNA expression levels of CD44 in the established SW480 tumors were measured by qRT-PCR using specific primers. Asterisk denotes statistical significance

CRC development is compatible with the CSCs concept, whereby a small cellular sub-populations within tumors (i.e., CSCs) has accentuated tumorigenic potential that initiates and maintains tumor development [22]. Thus, we hypothesized that the tumorigenic effects presented in mutant p53-expressing colorectal cell lines might be due to an increased CSCs sub-populations. It was reported that three bona fide markers for cancerous colon stem cells; CD44, Lgr5, and ALDH are highly expressed during the progression to carcinoma [19] and are associated with shorter time to disease recurrence [1]. Accordingly, we next examined whether the mutant p53-expressing colorectal cell lines also harbor larger Lgr5^Br^, CD44^Br^, and ALDH^Br^ sub-populationss. The RKO isogenic cell lines were immuno- stained with anti-Lgr5 antibody followed by evaluation of the size of Lgr5^Br^ sub-populations by hnageStream X analysis (Fig. 2a, b). The results indicate on a significantly larger Lgr5^Br^ cell sub-populations in mutant p53-expressing RKO cells (RKO+/m) compared with both RKO+/+ and RKO −/− cells (Fig. 2a, b). Thus, these data suggest that mutant p53 augments the Lgr5^Br^ sub-population.

Growing body of evidence suggest that Lgr5^+^ cells extracted from murine intestine have the potential to create an in vitro 3-dimensional crypt-villus structure organoids that are enriched with stem cells and fully resemble the intestine [23–25]. To determine whether mutant p53 affects Lgr5^Br^ levels also in a more physiological system, we examined organoids that were established from intestinal epithelial cells extracted from wild-type p53 (+/+), p53 knock-out (−/−), and p53 heterozygous mice-expressing mutant p53^R172H^ (the mouse equivalent to human p53^R175H^) and wild-type p53 alleles (+/m) [15]. Our analysis indicate that organoids that were generated from heterozygous mice displayed significantly increased Lgr5 transcription compared with organoids generated from wild-type or p53 knock-out mice (Fig. 2c). These results are in accordance with the results obtain from RKO cell lines and together suggest that mutant p53 induces Lgr5 levels in intestinal cells.

Next, to assess the size of RKO cellular sub-populations highly expressing CD44 (CD44^Br^), we immuno-stained the RKO cells with anti-CD44 antibody, followed by FACS analysis. As indicated in Fig. 2d, mutant p53^R248w^ knock-in RKO cells (RKO+/m) exhibited larger CD44^Br^ sub-populations compared with the wild-type p53 (RKO+/+) and knock-out (RKO−/−) counterparts. To corroborate these data, we immuno-stained the established SW480 cells with anti-CD44 antibody and analyzed CD44 levels by the ImageStream X instrument. In accordance with RKO cells, knocking-down the endogenous mutant p53^R273H,p309S^ (shp53) in SW480 cell line lead to reduction in the size of CD44^Br^ sub-populations (Fig. 2e–g). Furthermore, we measured the mRNA expression levels of CD44 in tumors that were generated upon injection of the established SW480 shCon and shp53 cell lines into immuno-deficient mice (Fig. 1c, d). The obtained results indicate on lower expression of CD44 in tumors generated from mutant p53 knock-down cells (shp53) compared with those endogenously expressing mutant p53 (shCon) (Fig. 2h). In all, these results suggest a positive correlation between mutant p53 and CD44 expression in CRC.

**Fig. 3:**
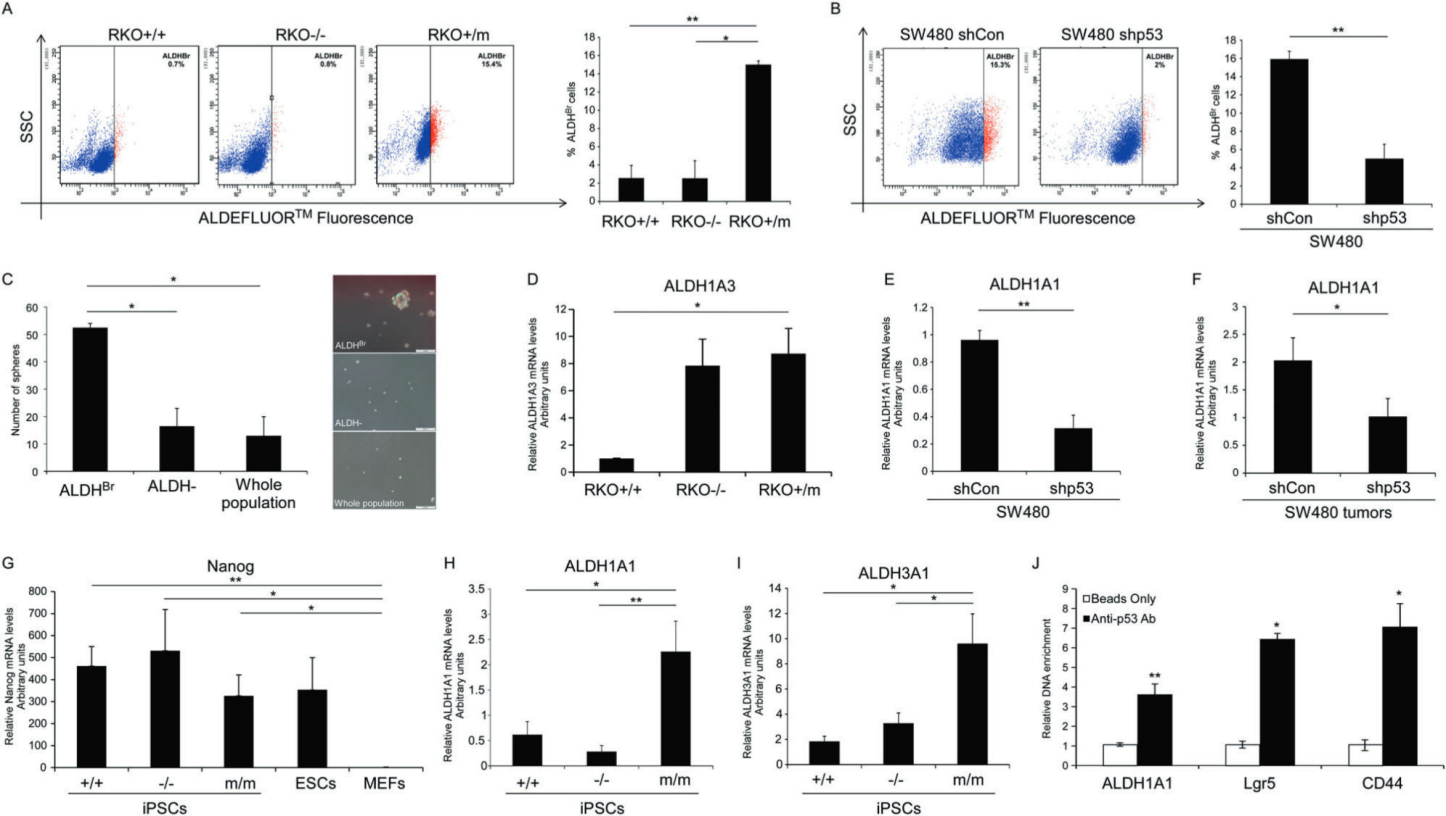
Mutant p53 induces larger ALDH^Br^ sub-populations. The RKO isogenic cell lines **(a)** and established shCon and shp53 SW480 cells **(b)** were subjected to ALDH activity assay and the size of ALDH^Br^ sub-populations was measured by FACS. Left panels show dot plots of representative experiment indicating on percentage of ALDH^Br^ cells. Right panels show graphs of averaged percentage of ALDH^Br^ cells obtained from three experiments. Error bars represent SE. **c** SW480 cell line endogenously expressing mutant p53^R273H,p309S^ was sorted by FACS according to ALDH activity levels. Then, the sorted populations were incubated in suspension under serum free spheroids promoting media. Following 2 weeks spheroids were counted via microscopic examination. Graph represents an averaged number of spheres detected in three experiments. Right panels are photos of representative fields, **d-f** mRNA expression levels of ALDH1A3 in RKO cell lines **(d),** ALDH1A1 in SW480 cell lines **(e),** and ALDH1A1 in the established SW480 tumors **(f)** were measured by qRT-PCR using specific primers, **g-i** MEFs that were extracted from wild-type p53 mice (+/+), p53 KO mice (−/−), and mutant p53^R172H^ knock-in mice (m/m) were subjected to standard reprogramming protocol [31] followed by measurement of mRNA expression levels of Nanog **g,** ALDH1A1 **h** and ALDH3A1 **i** by qRT-PCR using specific primers, **j** ChIP analysis of SW480 cells. Endogenous mutant p53 protein was immunoprecipitated using p53-specific antibody (anti-p53 Ab). Empty beads were used as a negative control (Beads only). qRT-PCR was performed using specific primers directed to ALDH1A1, CD44 and Lgr5 promoters. Values were normalized to 1% input. Results are average of three experiments. Error bars represent SE. Asterisk denotes statistical significance

Finally, we examined the effect of mutant p53 on ALDH levels in the various colorectal cancer cell lines and tumors. ALDH is a detoxifying enzyme that serves as a main marker for colorectal CSCs [19]. Notably, it mediates drug-resistance activities [26] and its high levels predict poor prognosis and cancer relapse [27, 28]. Thus, next we examined the size of the cell sub-populations that displays high-ALDH activity (ALDH^Br^) within the RKO isogenic cell lines. We found that mutant p53^R248w^-expressing cells (RKO+/m) display larger ALDH^Br^ sub-populations compared with their wild-type p53 and p53 KO counterparts (RKO+/+ and RKO−/−, respectively) (Fig. 3a). We further estimated the size of ALDH^Br^ sub-populations in the established SW480 cell lines, and found that shp53 cells harbor significantly reduced ALDH^Br^ sub-populations compared with shCon cells (Fig. 3b). This suggests that mutant p53 mediates the expansion of the ALDH^Br^ sub-populations within CRC cell lines.

A typical feature of CSCs is their ability to form floating spheres in culture [26, 29]. To validate that the ALDH^Br^ sub-populations is indeed enriched with CSCs, we measured their ability to generate spheroids in vitro. Therefore, using FACS we isolated SW480 shCon cells with either high or low ALDH activity, and then measured ALDH1A1 mRNA expression levels to validate the efficiency of sorting. Indeed, ALDH1A1 levels were found to be higher in ALDH^Br^ sub-populations compared with the ALDH negative (ALDH—) sub-populations and the whole cell line population (Supplementary Figure 4). The isolated cellular sub-populationss were cultured in suspension, in serum free media optimized for spheroids maintenance. Following 2 weeks of cultivation the ALDH^Br^ cells gave rise to a markedly larger number of typical floating spheres (Fig. 3c). This suggests that within the general mutant p53-expressing SW480 cell line population ALDH^Br^ cells possess self-renewal capacity, and constitute the CSCs compartment.

Next, we aimed to examine whether ALDH mRNA expression is regulated by mutant p53. ALDH1A1, ALDH 1 A3, and ALDH3A1 were suggested to have an important functional role in CSCs [26]. Accordingly, we evaluated the mRNA expression levels of ALDH1A1 and ALDH 1 A3 in the SW480 and RKO cellular systems, and found that both members are expressed in both cellular systems. Notably, while ALDH1A1 levels were increased in SW480 shCon cells compared with shp53 cells (Fig. 3e), ALDH1A3 expression was higher in RKO+/m and RKO *—/—* cells compared with wild-type p53 (RKO+/+) counterparts (Fig. 3d). To further validate that ALDH induction could be dependent on mutant p53 GOF we examined the mRNA levels of ALDH genes in the established HI299 cells (Supplementary Figure 5). Higher levels of ALDH1A1 and ALDH1A3 were detected in mutant p53^R175H^-expressing cells compared with p53 null cells, suggesting that the elevated levels of ALDH genes in HI299 is mediated by mutant p53 GOF. Finally, we measured the mRNA expression levels of ALDH1A1 in tumors that were generated upon injection of the established SW480 into nude mice (Fig. 1c, d). The results indicate on elevated expression of ALDH1A1 in tumors generated from mutant p53-expressing cells (shCon) compared with tumors derived from SW480-expressing shp53 cells (Fig. 3f). This finding further supports the positive correlation between mutant p53 and ALDH1A1 expression observed in our in vitro models. All in all, these data suggest that mutant p53-expressing CRC cell lines possess larger cellular sub-populations that display high-ALDH activity, thus suggesting larger CSCs sub-populations that may account for their higher tumorigenic potential and drug resistance.

Next, we aimed to exclude the possibility that the results described above pertaining the induced ALDH levels in mutant p53-expressing cell lines and their stable shp53 derivatives are due to additional genetic modifications that may arise following prolonged culturing. Therefore, we transiently knocked-down mutant p53^R273H,P309S^ from SW480 cells using specific siRNA oligonucleotides (Supplementary Figure 6A) and measured the size of ALDH^Br^ sub-populations by FACS (Supplementary Figure 6B). The results indicated that transient silencing of mutant pS3^R273H,P309S^ (p53i) reduced the size of ALDH^Br^ sub-populations within the original SW480 cell line (siCon). To further assess whether the endogenous mutant p53^R273H,P309S^ expressed in the SW480 cell line mediates the induction of ALDH1A1 expression, we adopted CRISPR/Cas9 technology to knock-out mutant p53^R273H,P309S^ expression. To this end, we established two CRISPR-derived cell lines; CRISPR control, which expresses the endogenous mutant p53^R273H,p309S^ and CRISPR p53 cell line, in which mutant p53^R273H,p309S^ was knocked-out (Supplementary Figure 7A). Consistent with our p53 knock-down experiments using both p53-specific shRNA and p53-specific siRNA, CRISPR p53 cells exhibited significantly reduced expression of ALDH1A1 (Supplementary Figure 7B), suggesting that mutant p53^R273H,P309S^ induces ALDH1A1 expression. In all, the results obtained from three independent mutant P53^R273H,P309S^ silencing systems (i.e., shRNA, siRNA and CRISPR/Cas9) showed reduction in ALDH1A1 mRNA expression and diminished ALDH^Br^ sub-populations, thereby supporting the conclusion that mutant p53 mediates ALDH induction.

It is suggested that the origin of CSCs might be dedifferentiation of somatic cells [16], a process that is represented by cellular reprogramming [30]. Moreover, we previously found that reprogramming of mutant p53- expressing MEFs lead to the generation of malignant iPSCs that can be referred as CSCs [17]. Thus, we next examined whether higher ALDH levels can be detected in mutant p53-expressing iPSCs. MEFs that were extracted from wild-type p53 mice (+/+), p53 KO mice (—/—) and mutant p53^R172H^ KI mice (m/m) were subjected to a standard reprogramming protocol [31]. As a first step Nanog mRNA expression levels were examined to validate the sternness of the generated iPSCs (Fig. 3g). The Nanog expression levels were comparable to those of embryonic stem cells (ESCs) and significantly higher than in MEFs, indicating successful reprogramming (Fig. 3g). ALDH1A1 and ALDH3A1 expression levels were markedly elevated in mutant p53-expressing iPSCs, compared with WTp53 (+/+) and p53 KO (—/—) iPSCs (Fig. 3h, i, respectively). This indicates that mutant p53 enhances ALDH expression in iPSCs, suggesting a positive correlation between mutant p53, ALDH levels and the generation of malignant CSCs.

Finally, to further decipher the mechanism underlying mutant p53-dependent induction of ALDH1A1 we first examined whether mutant p53-dependent induction of ALDH1A1 expression results from transcriptional activation of ALDH1A1 promoter. For that purpose, genomic fragment of 1 kb of ALDH1A1 promoter was fused to luciferase reporter and the vector was introduced into the established shCon and shp53 SW480 cell lines. As depicted in Supplementary Figure 8, mutant p5 3^R273H,P3()9S^-expressing cells showed higher luciferase activity than shp53 cells. This finding suggests that mutant p53 induces ALDH1A1 expression by activating its promoter. Next, we examined whether the induction of the CSCs markers involves the interaction of mutant p53 with their promoters. To this end, we performed chromatin immuno-precipitation (ChIP) assay on SW480-expressing p53^R273H,P309S^ and SK-BR-3 cell line, expressing mutant p53^R175H^. We detected enrichment of ALDH1A1, CD44, and Lgr5 promoter sequences upon immune-precipitated mutant p53 using specific antibody (Fig. 3j; Supplementary Figure 9). These results suggest that the enhanced levels of ALDH1A1, CD44, and Lgr5 observed in mutant p53-expressing cells may be mediated by mutant p53 binding to ALDH1A1, CD44, and Lgr5 promoters.

### Mutant p53-dependent cell death resistance is mediated by ALDH

**Fig. 4:**
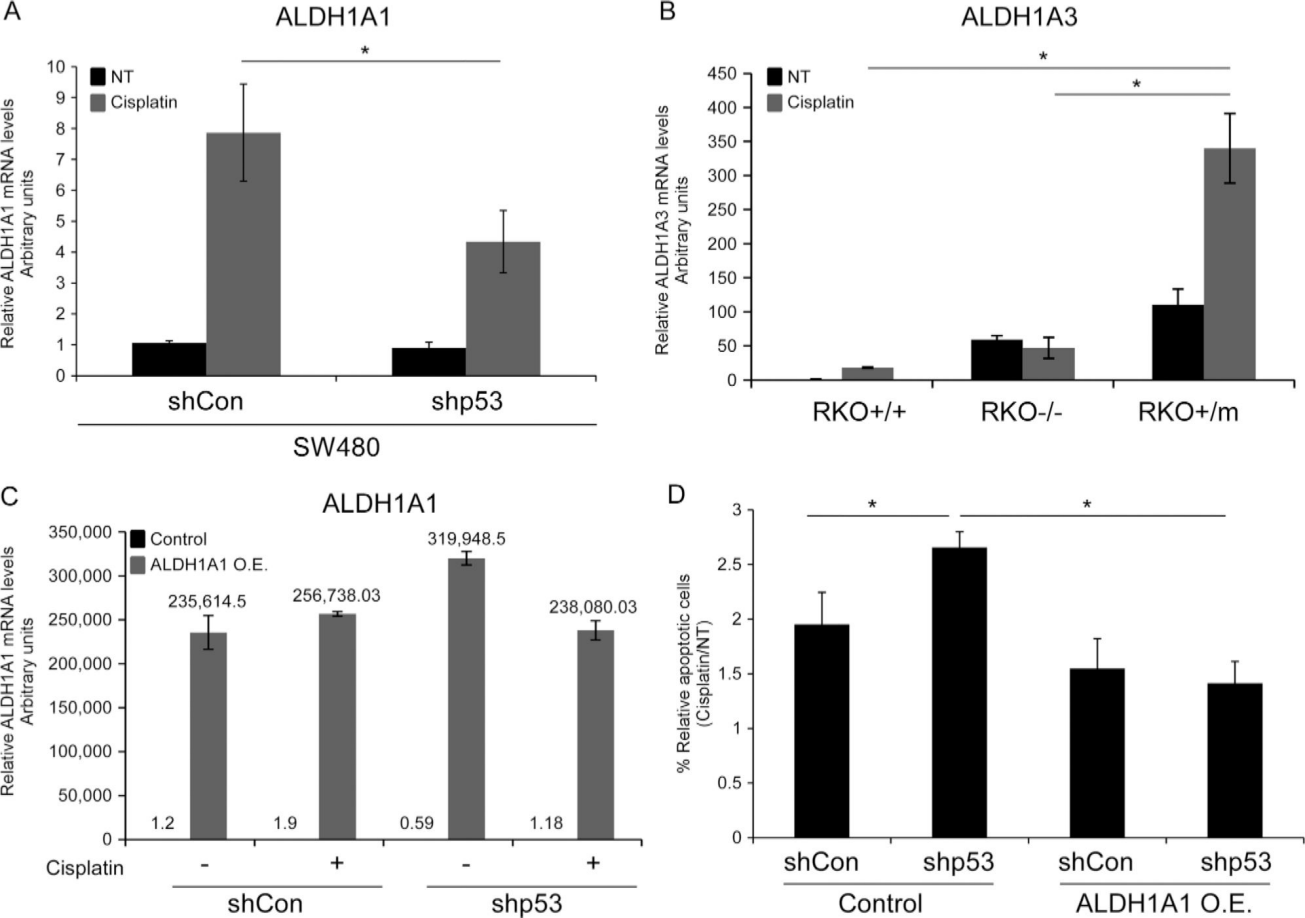
Mutant p53-dependent chemotherapy resistance is mediated by ALDH. **a, b** the established shCon and shp53 SW480 and the RKO isogenic cell lines were treated with cisplatin (2.5 μg/ml) for 72 h. mRNA levels of ALDH1A1 **a** and ALDH1A3 **b** were measured in SW480 and RKO, respectively, by qRT-PCR using specific primers, **c**, **d** The established shCon and shp53 SW480-expressing mutant p53^R273H,F309S^ were transiently transfected with pcDNA3-HA-ADH to over-express ALDH1A1. Following 24 h, cells were treated with cisplatin (2.5 μg/ml) for additional 72 h. Then, cells were collected and subjected to AnnexinV staining and to ImageStream X analysis. Right panel indicates on percentage of dead cells. **c** mRNA levels of ALDH1A1 indicating on successful transfection, resulting in ALDH1A1 over-expression in the cells. **d** Graph indicating on percentage of apoptotic cells upon cisplatin treatment, normalized to NT cells. All graphs represent an average of three experiments. Error bars represent SE. Asterisk denotes statistical significance. *OE* over-expression. For more representative photos obtained by ImageStream X see Supplementary Figure 13E

It was reported that CSCs may facilitate drug resistance by activating ALDH [26]. Notably we observed that mutant p53 endows CRC tumor cells with drug resistance (Fig. 1e, f; Supplementary Figure 3) and induces increased ALDH expression and activity. Therefore, we hypothesized that ALDH is involved in the mutant p53-induced drug resistance. To examine this hypothesis, the established SW480 and RKO isogenic cell lines with different p53 status were treated with cisplatin and the mRNA levels of ALDH1A1 and ALDH1A3 were estimated. Chemotherapy treatment resulted in marked induction of ALDH1A1 and ALDH1A3 expression levels (Fig. 4a, b). Notably, ALDH1A1 and ALDH1A3 expression reached to higher levels in mutant p53-expressing cells than in mutant p53-deficient cells, demonstrating the contribution of ALDH for mutant p53- mediated chemoresistance. To further examine whether ALDH1A1 promotes the mutant p53-dependent cell death resistance, we over-expressed ALDH1A1 in the established SW480 cell lines by transient transfection (Fig. 4c) followed by treatment with cisplatin and AnnexinV staining. Then, to assess apoptosis levels, cells were analyzed by ImageStream X. Our analysis revealed that ALDH1A1 over-expression significantly inhibited apoptosis in SW480 cells (Fig. 4d), suggesting that ALDH1A1 is involved in mutant p53-mediated chemoresistance of SW480 cells.

### Human colorectal carcinomas harboring p53 missense mutations display elevated proteins levels of ALDH and CD44

**Fig. 5:**
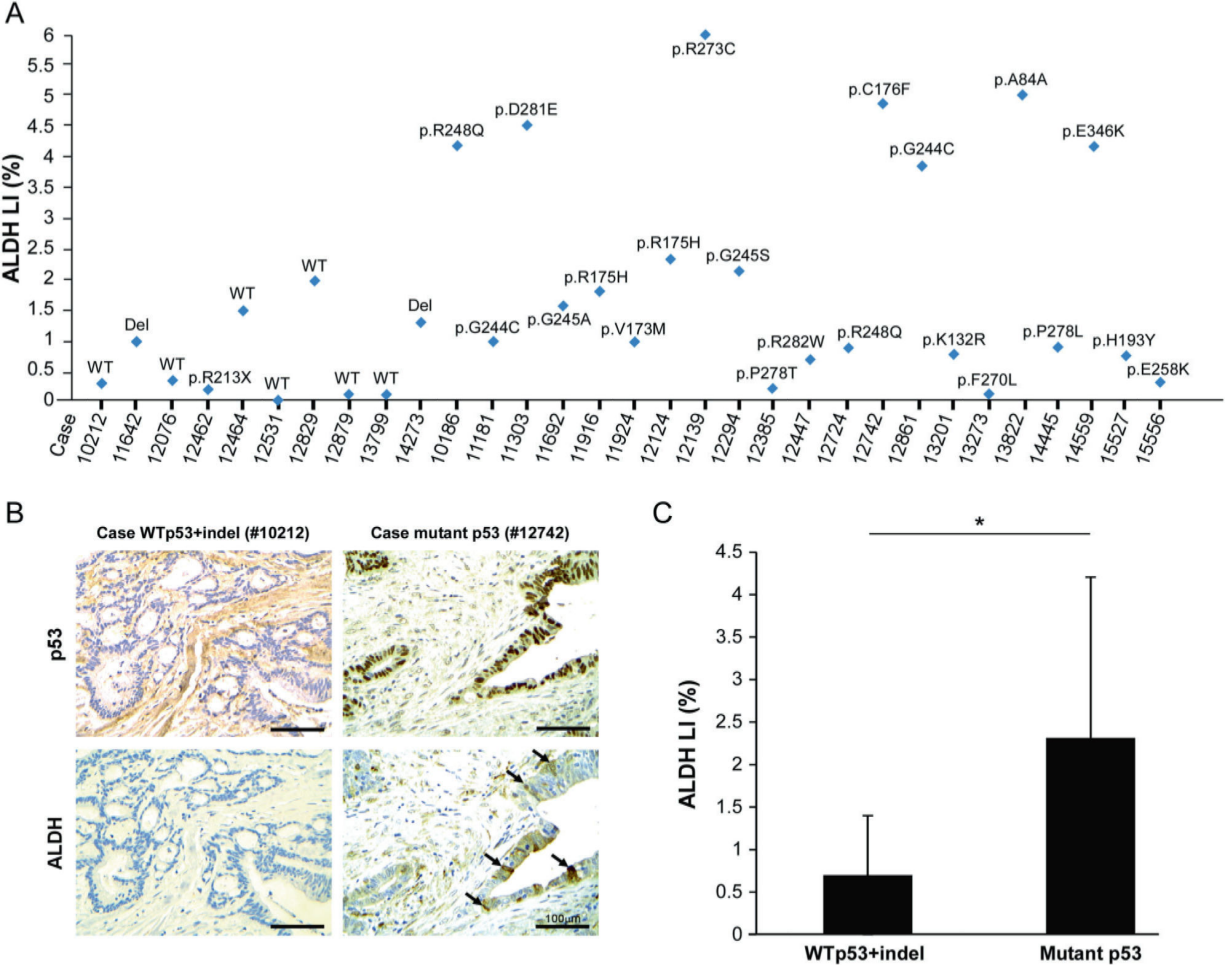
Mutant p53 correlates with elevated ALDH levels in patients with colorectal carcinoma. Tumor biopsies obtained from sporadic colorectal carcinoma patients were immuno-stained for ALDH and p53, and underwent *TP53* sequencing, a Diagrammatic presentation of the status of ALDHA1 along with the *TP53* sequencing data for every individual case. *Π* labeling index, b Representative photos of ALDH and p53 staining showing increased ALDH levels in a mutant p53 case compared to a WTp53 one. Arrows denote positive ALDH staining. Scale bar: 100 μm. **c** Cases with missense mutations of p53 exhibit increased ALDH levels vs. WTp53 + indel ones. Asterisk denotes statistical significance (*p*-value < 0.05)

Human CRC is characterized with frequent recurrence following conventional therapy. It is well-accepted that the CSCs entity within tumors underpins chemoresistance and tumor relapse [1]. In addition, it was found that CRC exhibit high frequency of p53 mutations. Based on these notions we set out to investigate whether our observations that mutant p53 GOF is correlated with high levels of CSCs markers also applies to human CRC biopsies. Therefore, paraffin- embedded tissues of human CRC tumors biopsies obtained from either sporadic colorectal carcinoma patients or colitis- associated colorectal carcinoma patients were subjected to p53 gene sequencing along with immunohistochemistry analysis of CD44, ALDH, and p53. Since p53 missense mutations endow oncogenic GOF activities [10], for the analysis we divided the tumors to two groups; WTp53/indel and p53 missense mutations. Strikingly, we observed that ALDH levels were markedly higher in both sporadic and colitis-associated CRC tumors expressing p53 missense mutations compared with tumors expressing WTp53/indel mutations (Fig. 5a–c; Supplementary Figure 10A–B). Additionally, we could detect positive correlation (Pearson correlation coefficient is ρ = 0.53) between p53 and ALDH labeling index in colitis-associated colorectal carcinoma samples (Supplementary Figure 10c). As intense p53 staining in tumor biopsies usually indicates mutant p53 expression, this result might suggest a positive correlation between high-ALDH levels and mutant p53 expression. In line with our finding pertaining ALDH expression in CRC tumors our analysis indicated that tumor samples expressing p53 missense mutations tend to exhibit higher levels of CD44 (Supplementary Figure 11).

Interestingly, when we analyzed patients’ disease characteristics, we revealed that tumors expressing p53 missense mutations are associated with more aggressive cancer. Specifically, tumors expressing p53 missense mutations are more invasive (Supplementary Figure 12a), and patient harboring tumors expressing p53 missense mutations exhibited distant metastasis in higher frequency (Supplementary Figure 12b) and in more regional lymph nodes (Supplementary Figure 12C), compared with that observed in patients harboring WTp53/indel-expressing tumors. In general tumors expressing p53 missense mutations were defined as grade ΙΠ and IV in higher frequency than WTp53/indel-expressing tumors (Supplementary Figure 12D). This was even more pronounced in patient harboring tumors expressing both p53 missense mutations and high levels of ALDH (>1.5 LI) compared with those expressing p53 missense mutations and lower levels of ALDH LI (Supplementary Figure 12E–H). Finally, the survival after adjuvant therapy of patients that harbor tumors expressing p53 missense mutations and higher ALDH (>1.5 LI) was in lower frequency than in patient harboring tumors expressing p53 missense mutations and lower ALDH (<1.5 LI), implying that mutant p53/high-ALDH tumors are more chemoresistant (Supplementary Figure 12I). In all, in accordance with our in vitro study, these data suggest that mutant p53 expression in human CRC tumors positively correlates with an elevated expression of ALDH and CD44, bona fide CSCs markers, which might explain the higher disease aggressiveness.

## Discussion

A prevalent model mechanism for cancer progression in the recent years is the hierarchy model, which predicts that only a small subset of cells within tumors, termed tumor- initiating cells or CSCs has the ability to proliferate and propagate the tumor, as well as to differentiate into various lineages and generate the tumor heterogeneity [5]. CSCs can evolve from various cellular origins that underwent genetic aberrations; including normal adult stem cells, progenitor cells, or following dedifferentiation of somatic cells. Regardless of cell origin, all CSCs carry aberrant genetic setup [6]. Therefore, to maintain normal homeostasis, cells must be tightly regulated. The tumorsuppressor p53 guards the genome, and ensures the genomic integrity of cells. Hence, while wild-type p53 usually serves as a barrier to CSCs formation [6], it appears that mutant p53 GOF promotes acquisition of CSCs features [32], Interestingly, many of the well-accepted mutant p53 GOF activities [11] are shared with CSCs characteristics. For example, CSCs are known to have high mitotic activity with unregulated self-renewal capacity, to give rise to macroscopic metastases, to possess inherent drug resistance, and to generate tumor heterogeneity [33]. These features are also attributed to mutant p53 GOF activities, where mutant p53 mediates extensive proliferation, metastases formation, drug resistance [11] and gives rise to undifferentiated tumors [16]. Interestingly, several studies suggest that CRC behaves according to the CSCs hypothesis [34], Given all, in this study we investigated whether mutant p53 enhances CRC tumorigenesis by expanding the CSC sub-populations within CRC tumors.

It is accepted that p53 is frequently mutated in CRC, driving tumor aggressiveness [3]. Since it is accepted that the various p53 mutations mediates distinct oncogenic GOF activities [35], in this study we utilized different cell Unes that express distinct p53 missense mutation to study the effects of these mutant p53 proteins on CSC sub-populationss. These missense mutations belong to two p53 mutations sub-groups that define p53 mutation type according to their impact on the DBD folding; “DNA-con- tact mutations” (R248, R273), and the “p53 conformational mutations” (R175) [14].

As a first step we examined whether mutant p53 confers CRC cell lines with more aggressive tumorigenesis in vivo. Indeed, we found that knocking-down mutant p53 expression significantly reduced the capability of the SW480 cells to generate tumors in vivo (Fig. 1b–d). This observation is in accordance with the well-accepted concept that mutant p53 expression facilitates tumorigenesis in vivo [11]. Moreover, it also supports the newly evolved notion that tumor cells are addicted to mutant p53 and fail to develop tumors in its absence [36]. Of note, pathological examination of the generated tumors determined that regardless of the p53 status in SW480, the tumors displayed similar morphologic features including high mitotic rate, mild to moderate multifocal infiltration of mononuclear cells, necrosis and fibrosis (Supplementary Figure 2e). This observation might suggest that the differences in tumor size stem from increased tumor initiation capacity of mutant p53-expressing cells.

To examine whether mutant p53 enhances the expansion of the CSCs sub-populations, we focused on studying mutant p53 regulation of three main colorectal CSCs markers; Lgr5, CD44, and ALDH [1], We detected Lgr5^Br^, CD44^Br^, and ALDH^Br^ sub-populations within the examined colorectal cell lines, and found that they were larger in mutant p53-expressing cells compared with WTp53 or p53 non- producing cells (Figs. 2, 3). Notably, we found that RKO —/— cells possess larger CD44^Br^ population compared with RKO+/+ cells, yet, it is markedly smaller than the CD44^Br^ sub-populations present in RKO+/m cells (Fig. 2d). This observation is consistent with Godar et al. that reported on inhibitory effect of WTp53 on CD44 expression in breast cancer cells [37]. Nevertheless, our data demonstrate that mutant p53-expressing RKO cells possess significantly larger CD44^Br^ sub-populations than RKO—/—, suggesting that besides loss of function, mutant p53 displays GOF activity. In contrast, we found that the expression of ALDH1 A3 in RKO—/— is similar to that of RKO+/m (Fig. 3d). Therefore it seems that in RKO cells mutant p53^R248w^ acts in a loss of function manner in inducing ALDH 1 A3. Yet, analysis of ALDH activity in RKO cellular system (Fig. 3a) indicated a larger sub-populations of highly activated ALDH in RKO+/m cells compared with both RKO +/+and RKO—/— cells, suggesting a mutant p53 GOF. This discrepancy in the manner of mutant p53 function implies on possible additional level of regulation of mutant p53 on ALDH.

Moreover, utilizing a more physiological model such as intestinal organoids that represent normal intestinal tissue, we found that Lgr5 transcription was enhanced in organoids derived from mutant p53^R172H^-expressing mice compared with WTp53 and p53 null mice (Fig. 2c). These findings imply that mutant p53 exerts a negative effect on intestinal tissue homeostasis. Aiming to reveal the mechanism by which mutant p53-induced the expression of the CSCs markers, we found that mutant p53^R273H,P309S^ enhanced the activity of ALDH1A1 promoter (Supplementary Figure 8), and bound the promoter sequences of ALDH, CD44, and Lgr5 genes (Fig. 3j). These results suggest that the enhanced expression of ALDH1A1, CD44, and Lgr5 in the examined mutant p53-expressing cells might be mediated by mutant p53 binding to their promoters. Notably, it is well-accepted that DNA binding of mutant p53 to p53 responsive elements is compromised [10]. Nevertheless, mutant p53 may regulate genes transcription indirectly via binding and stabilizing other transcription factors [11]. Therefore, it is plausible that mutant p53 regulation of ALDH1A1, CD44, and Lgr5 involves interaction with additional transcription factors, rather than direct binding to ALDH1A1, CD44, and Lgr5 chromatin. To further reveal the potential transcription factors that are involved in ALDH1A1, Lgr5, and CD44 induction by mutant p53, we used the Matinspector tool (Genomatix genome analyzer) to identify putative transcription factor binding sites in ALDH1A1, Lgr5, and CD44 sequences (Supplementary Table 1). Interestingly, our analysis demonstrated that one transcription factor binding site, corresponding to the transcription factor family termed SORY, (SOX/SRY-sex/testis determining and related HMG box factors) is common in the three gene sequences. Thus, this family of transcription factors may serve as potential candidates for mediating mutant p53 function (Koifinan et al. in preparation). However, the exact mechanism by which mutant p53 induces the expression of ALDH1A1, Lgr5, and CD44 remains to be investigated. One of the well-known mutant p53 activities is enhancing tumor chemotherapy resistance [10, 11]. Indeed, we demonstrated that in our cellular systems mutant p53 attenuates apoptosis in response to chemotherapy (Fig. 1e, f; Supplementary Figure 3). Since chemotherapy resistance is a well-accepted characteristic of both CSCs and mutant p53-expressing cells, it raised the possibility that the observed mutant p53-dependent chemotherapy resistance is mediated by enhanced CSCs features of tumor cells. Indeed, when ALDH1A1 was over-expressed in SW480 cells we observed decreased apoptosis, indicating on ALDH1A1 involvement in mediating mutant p53-dependent chemotherapy resistance (Fig. 4c, d). Furthermore, chemotherapy treatment resulted in induction of ALDH1 expression levels that was markedly higher in mutant p53 cells compared with wild-type p53 or p53 non-producers (Fig. 4a, b), indicating on the important role of ALDH1 in mediating mutant p53-dependent chemotherapy resistance. These data is supported by a recent clinical study that examined the expression of known CSCs markers in rectal cancer patients, and suggested that their expression is elevated upon radiotherapy, while high ALDH1 predicts poor prognosis and cancer recurrence [27]. Interestingly, while in basal levels ALDH1A3 is induced by mutant p53 loss of function manner (Fig. 3d and Fig. 4b); cisplatin treatment modifies the mode of mutant p53 activity, resulting in a GOF activity (Fig. 4b).

CSCs can emerge from somatic cells that underwent genetic aberrations due to dedifferentiation process. Previously, we showed that mutant p53-expressing MEFs that underwent reprogramming, generated iPSCs with malignant phenotype in vivo, thus can be referred as CSCs [17]. Here, we found that these aberrant iPSCs also express higher levels of ALDH1A1 and ALDH3A1 than the wild-type p53 and p53 KO counterparts (Fig. 3g–i); supporting our observation that mutant p53 induces ALDH expression and acquires cells with malignant CSCs phenotype.

Finally, analysis of human CRC biopsies revealed higher expression of ALDH1 and CD44 in tumors expressing p53 missense mutations compared with wild-type p53 or p53 indel mutation-expressing tumors, which further emphasizes the importance of our observations for human CRC patients.

Of note, Zeilstra et al. reported that CD44 expression is independent of p53 status in human colorectal cancer [38]. The discrepancy from our observation may stem from the fact that in their analysis Zeilstra et al. included tumors with all p53 mutations types in the same group, whereas, we divided the examined CRC tumors to two groups: (1) tumors expressing p53 missense mutations that are known for their oncogenic functions and (2) tumors expressing WTp53 or p53 insertion/deletion mutations that usually exert loss of function. Possibly, our observed phenotype that ALDH and CD44 expression is increased in the first group of tumors is related particularly to missense mutations and not to p53 deletions (Fig. 5; Supplementary Figures 10–11). This supports our conclusion that mutant p53 induces colorectal CSCs markers in a GOF manner.

Interestingly, various p53 mutations types are correlated with distinct ALDH levels in tumors of different patients. However, this distribution was not found necessarily related to the specific mutant p53 type. Additionally, we could also observe that the same p53 mutation type, expressed in tumors of different patients, might have different effect on ALDH levels, suggesting that the genetic and environmental background of patients could also contribute to ALDH levels in the tumors. These observations are in line with the notion that various p53 mutations may have different oncogenic GOF activities [35]. Nevertheless, statistical analysis of human sections revealed that tumors expressing p53 missense mutations also express significantly higher *(p <* 0.05) ALDH levels compared with WTp53/indel tumors (Fig. 5c). Thus, we concluded that the expression of various p53 mutations potentially correlates with high-ALDH levels in human CRC. Of note, the IARC database analysis for p53 mutations in human colorectal cancers revealed that the most frequently mutated positions are 175, 248, and 273. Interestingly, these are the mutations that we focused on in this study and the ones showing the highest ALDH LI in the analysis of human sections. Thus, we concluded that the expression of various p53 mutations, and specifically, the most frequent in human CRC, potentially correlates with high-ALDH levels in human CRC.

Furthermore, correlation between the tumor stage, p53 status and ALDH1 expression revealed that tumors expressing p53 missense mutations and high-ALDH LI are more aggressive. Accordingly, patients harboring such tumors showed reduced survival upon adjuvant therapy, implying that mutant p53/high-ALDH tumors are more chemoresistant. Of note, the cohort of patients harboring mutant p53-expressing tumors that received chemotherapy and was available for our analysis is rather small *(n =* 12), and their background is different. Therefore, our analysis imply on a possible link between mutant p53, ALDH1, and patients’ chemoresistance, but further clinical investigation is needed to validate the statistical significance of the study.

Our hypothesis that mutant p53 facilitates CRC development by enhancing the expansion of colorectal CSCs sub-populations is supported by two recent studies that suggested that mutant p53 promotes stem cell-like features of lung, breast [39], and osteosarcoma [40] tumor cells.

In sum, our findings indicate a positive correlation between various p53 missense mutations and bona fide colorectal CSCs markers, which was corroborated in several in vitro colorectal cellular systems (SW480 and RKO), in vivo tumor models, and human biopsies. These findings provide novel insights pertaining the mechanism by which mutant p53 enhances CRC development, which involves the expansion of CSCs sub-populations within CRC tumors, and underscore the importance of targeting these sub-populationss for CRC therapy.

## Materials and methods

### Vectors

The pRetroSuper-p53 shRNA-Blast that was used for silencing p53 (sequence: 5′-GACTCCAGTGGTAATC-TAC-3′), and the pRetroSuper-shmNOXA-Blast (sequence: 5′-AAGGGACATCTGTACTTCTGG-3′) that was used as shRNA control were kindly provided by Dr. Doron Ginsberg (Bar-Ban University, Israel). The pRetroSuper-sh-LacZ-puro (sequence: 5′-GTGACCAGCGAATACCTG-3′) was kindly provided by Prof. Reuven Agami. The pWZL-blast-mutant p53^R175H^ over-expression vector was constructed as previously described [41]. pcDNA3-HA-ADH that was used for ALDH1A1 over-expression, was a gift from Steven Johnson (Addgene plasmid # 11610). pX330 p53 vector that was used to knock-out human mutant p53 by CRISPR/Cas9 system (gRNA sequence: 5′-CCATTGTTCAATATCGTCCG-3’) was kindly provided by Dr. Jacob Hanna. pCLuc-Basic2 vector harboring ALDH1A1 promoter was cloned by restriction-free (RF) cloning, in which lkb of ALDH1A1 promoter sequence was isolated from genomic DNA using the following primers: F: 5′-GGATCGGGAGATCTTGGAATTCTGCA-GATAcccatgtaggagttctcttgtg-3′, R: 5′-CCTTAATATGCG AAGGATCCGAGCTCGGagctgctctggccactaaggcc-3′, followed by PCR product clean and secondary PCR using pCLuc-Basic2 vector (NEB) as a template.

### Transfections and retroviral infections

Mutant p53 stable knock-down or mutant p53^R175H^ over-expression was performed by stable infections, using ecotropic Phoenix-packaging cells as previously described [42]. p53 transient knock-down was conducted by cell transfection of specific small interfering RNA (siRNA) oligonucleotides against p53 using Dharmafect3 reagent (Dharmacon). siRNA ON-TARGETplus SMARTpool oligonucleotides were purchased from Dharmacon, including the following sequences: 5′-GAAAUUUGCGUGUGGA- GUA-3′; 5′-GUGCAGCUGUGGGUUGAUU-3′; 5′-GCA-GUCAGAUCCUAGCGUC-3′; 5′-GGAGAAUAUUUCACCCUUC-3′. Control LacZi sense sequence: 5’-GUGACCAGCGAAUACCUGU-3′. pCLuc-Basic2 vector harboring ALDH1A1 promoter, pCMV-GLuc, and CMV-GFP-expressing vectors were co-transfected to SW480 cells using FugeneHD reagent (Promega).

### Knock-out of mutant p53^R273H,P309S^ genes using CRISPR/Cas9

SW480 cells were transfected with pX330 vector harboring sgRNA specific to human p53 using FugeneHD reagent (Promega). After 48 h single cells were plated on 96 well plates and upon clone’s expansion, p53 expression levels were examined by western blot.

### Cell culture

All cell lines were cultured in a humidified incubator at 37° C and 5% C02 in medium supplemented with 10% fetal calf serum (FCS) and Pen/Strep solution (Biological industries). The human colon carcinoma cell line SW480 was maintained in DMEM (Biological industries). The human colon cancer isogenic RKO cell lines were kindly provided by Prof. Bert Vogelstein (The Johns Hopkins University, MD, USA) and maintained in McCoy’s 5A medium supplemented with 2mM L-glutamine. The non-small lung carcinoma cell line, NCI-H1299, was maintained in RPMI-1640. iPSCs were generated as previously described [31] and maintained on irradiated MEFs in ES medium [DMEM (Biological Industries) containing 15% FCS, 5 mg recombinant human LIF (Millipore), 1 mM glutamine (Biological Industries), 1% nonessential amino acids (Biological Industries), 0.1 mM β-mercaPtoethanol (Invitrogen), 60μg/ml penicillin, and 100 μg/ml streptomycin (Biological Industries)]. Intestinal organoids were generated as previously described [15]. SK-BR-3 breast cancer cells were maintained in RPMI-1640 medium. Mycoplasma test is being performed routinely.

### Spheroids formation

Six-well plates were covered with 95% poly(2-hydro-xyethyl methacrylate) (ployHEMA (Sigma-Aldrich)). Cells (4 × 10^4^ cells/well) were cultured in suspension in serum free spheroids promoting media (Insulin (5 μg/ml), hEGF (20ng/ml), βFGF (10ng/ml), LIF (5ng/ml), bovine serum albumin (BSA) (0.4%), Pen/Strep solution). Following 2 weeks of cultivation spheroid formation was assessed via microscopic examination (Olympus 1X51).

### Western blot

Cells were lysed in tris triton lysis buffer (50 mM Tris-Cl, 100 mM NaCl, 1% Triton X-100, 0.5% sodium deox-ycholate, and 0.1% SDS) supplemented with protease inhibitor mixture (EMD Millipore) for 20 min on ice. Extracts were analyzed for protein concentration by BCA protein assay (Thermo Scientific). For electrophoresis, 50 pg of protein extracts were dissolved in sample buffer (140 mM Tris (pH 6.8), 22.4% glycerol, 6% SDS, 10% β-mer-captoethanol, and 0.02% bromphenol blue) boiled and loaded on 10% polyacrylamide gels containing SDS. Proteins were transferred to nitrocellulose membranes at semidry conditions. The following antibodies were used: anti human p53 (DO-1) was kindly provided by Prof. Sir David Lane (Ninewells Hospital and Medical School, Dundee, Scotland); anti-GAPDH (Santa Cruz Biotechnology, USA); anti PARP-1 (Cell Signaling Technology). The protein-antibody complexes were detected by horseradish peroxidase-conjugated secondary antibodies and the ECL kit (Thermo Scientific), using the ChemiDoc MP imaging system (BIO-RAD).

### RNA isolation and quantitative real-time PCR

RNA isolation from cell lines and quantitative real-time PCR (qRT-PCR) was conducted as previously described [41]. RNA from tumor tissues was isolated using the Direct-zol RNA MiniPrep kit (Zymo Research). The specific primers that were designed for qRT-PCR are listed in Table 1. Human gene values were normalized to GAPDH, mouse gene values were normalized to HPRT, and human CD44 values obtained from tumor samples were normalized to 18S. qRT-PCR data is described in arbitrary units.

### Chromatin immuno-precipitation assay

ChIP was conducted as previously described [43]. The specific primers used for qRT-PCR are listed in Table 1.

### Reporter assay

Reporter assay was performed using the BioLux Gaussia and Cypridina Luciferase assay kit (NEB). SW480 cells (5 × 10^4^) were plated in 48-well plates in triplicate and 24 h later were transiently co-transfected with 480ng/well of pCLuc-Basic2 (Cypridina luciferase) harboring ALDH1A1 lkb promoter sequence, 10ng/well of pCMV-GLuc (Gaussia luciferase) and 10ng/well of CMV-GFP vector as controls for transfection efficiency. Luminescence values were estimated after 24 h. In order to estimate transfection efficacy, values of the pCLuc-Basic2-ALDHlAl were normalized to values of pCMV-GLuc.

### Apoptosis assay

Cells were analyzed for apoptosis using the AnnexinV-FLUOS Staining Kit (Roche). Apoptotic cells exposing phosphatidylserine on their surface were stained and subsequently detected by ImageStream X mark Π (Amnis Corp., part of EMD Millipore) using bright field and the 488 nm laser. At least 10,000 cells were gated for single cells using the area and aspect ratio features, and for focused cells using the Gradient RMS feature. Data were analyzed using the IDEAS 6.2; Amnis Corp. software. Annexin-V-positive cells were gated using the intensity (sum of the background-subtracted pixel values) and the Max Pixel (the largest value of the background-subtracted pixels) features. In addition, only cells with membrane staining were further gated using the Max Contour Position (the location of the contour in the cell that has the highest intensity concentration mapped to a number between 0 and 1, with 0 being the object center and 1 being the object perimeter). Cells were further gated using the circularity feature (deviation from circle of the object) and circular cells with high AnnexinV intensity were considered as apoptotic. The percentage of these cells of single, focused cells was calculated. Examples of cell images are shown in Supplementary Figure 13.

### ALDH activity

The ALDEFLUOR kit (StemCell Technologies) was used to measure the population with a high-ALDH enzymatic activity. Cells were collected using trypsin, and subjected to ALDEFLUOR assay according to the manufacturer’s protocol. The brightly fluorescent ALDH-expressing cells (ALDH positive cells) were detected in the green fluorescence channel (520–540 nm) of a LSRH flow-cytometer instrument (BD Biosciences). The gates were established using negative controls cells that stained with ALDEFLUOR and treated with 4-diethyl-aminobenzaldehyde an ALDH inhibitor, provided with the ALDE-FLUOR kit, according to the manufacturer instruction. Then, we gated the highest 15% of mutant p53-expressing cellular population and defined them as ALDH^Br^. The same gate was applied to all samples. Data was analyzed by using FACSDiva software (Version 6.1.3, BD Biosciences).

### Lgr5 and CD44 Immunostaining

Cells were seeded on 6 cm plates. The cells were washed with 2 ml of cold PBS and incubated with 1 ml of non-enzymatic cell dissociation solution for 5 min in 37 °C, followed by suspending in cell culture medium supplemented with 10% FCS. Cells (1.0 × 10^6^) were collected and pellets were re-suspended with 100 μΐ of FACS buffer (PBS 7.2 pH, 0.5% BSA, and 2 mM EDTA), and incubated either with anti-Lgr5 (Acris Antibodies Catalog No. TA400001) or with anti-CD44-APC (eBioscience, Catalog No. 17–044182) conjugated that recognizes all CD44 isoforms, for 10 min or 1 h, respectively, in the dark at 4 °C. Following washing and resuspending in FACS buffer, RKO, and SW480 cells were analyzed by ImageStream X mark Π (Amnis Corp., part of EMD Millipore) for Lgr5 or CD44 analysis, respectively. To evaluate staining intensity, a mask that defines the membrane by subtracting an eroded mask (Adaptive erode_77) from the general mask of the bright field image was created. Then, the mean pixel was calculated for this mask and a gate was set for the top 15% highest intensity of mutant p53-expressing cells. The same gate was applied to all samples. Additionally, RKO cells were, analyzed for CD44 staining by flow cytometry using LSRII flow-cytometer (BD Biosciences). Data was analyzed by using FACSDiva software (Version 6.1.3, BD Biosciences). For gate settings, cells were stained with isotype control antibody or secondary antibody only to establish nonspecific staining and define the positive regions. Then, the highest 15% of mutant p53-expressing cellular population were defined as CD44^Br^. The same gate was applied to all samples.

### Immunohistochemistry

For IHC analysis the following antibodies were employed: anti-p53 (D07, Santa Cruz), anti-ALDH recognizing 7–128 aa in ALDH1 which is highly similar in both ALDH1A1 and ALDH1A3 (BD Bioscience, Catalog No.611195) and anti-CD44 (Millipore). IHC was performed on paraffin-embedded tissues. Unmasking of the antigen retrieval was performed by heat-mediated antigen retrieval method in 10 mM citric acid (pH 6.0). The UltraVision LP Detection System was employed (#TL-060-HD, Thermo Scientific, Bioanalytica, Greece) according to the manufacturer’s instructions. For color development 3,3′-diaminobenzidine tetrahydrochloride (Sigma) was employed and hematoxylin was used as counterstain. Evaluation of p53 was performed as previously described [15, 44], The evaluation of ALDH was performed by counting the percentage of ALDHA1-positive cancer cells. CD44v6 evaluation was employed based on Zeilstra et al. [38]. An Overall Staining Score was employed by multiplying the intensity and the labeling index. The intensity was scored as follows: 0 for no reaction, 1 for faint staining, 2 for moderate staining, and 3 for intense staining. The labeling index was evaluated by counting the percentage of positive cells. Positive ALDH and CD44v6 immunostaining at the base of the colon crypts served as positive control. Three independent observers carried out slide examination, with minimal inter-observer variability.

### Mice

HsdHli:CDl-Foxnlnu Nude mice were used in this study (Harlan). Animal procedures were approved by the Institutional Animal Care and Use Committee of the Weizmann Institute of Science (14401114–1). Mice were monitored twice a week for tumor size.

### Human tissues and p53 sequencing

Primary tumor biopsies were obtained from the National Cancer Institute and the Mount Sinai School of Medicine. Patients’ CRC state was evaluated according to the TNM (Tumor, Node, Metastasis) staging system. The project was approved by the institutional review boards of the National Institutes of Health and the Mount Sinai School of Medicine (Office of Human Subjects Research approval number 3637). DNA extracted from tumor cell-enriched areas of each specimen was subjected to TP53 tagged amplicon Dlumina HiSeq 2000 sequencing [45].

### Statistical analysis

Statistical significance was evaluated using one-tailed unpaired Student’s *t* test. *p< 0.05, **/?<0.01. Statistical analyses of ALDH LI in human sporadic colorectal carcinoma sections were performed by one way ANOVA. Pearson correlation were performed using GraphPad Prism (GraphPad Software, San Diego, CA)

## Supplementary Material

Supplemental Material

## Figures and Tables

**Table 1:** Primers used for quantitative real-time PCR

Gene symbol	Forward primer (5′—3′)	Reverse primer (5′-3′)
Human ALDH1A1	GCACGCCAGACTTACCTGTC	CCTCCTCAGTTGCAGGATTAAAG
Human ALDH1A3	AGATACTTTGCAGGGTGGGC	GGGGGAAGTTCCATGGAGTG
Human GAPDH	ACCCACTCCTCCACCITTGA	CTGTTGCTGTAGCC AAATT CGT
Human Lgr5	CACCTCCTACCTAGACCTCAGT	CGCAAGACGTAACTCCTCCAG
Human p53	CCCAAGCAATGGATGATTTGA	GGCATTCTGGGAGCTTCATCT
Human CD44	TCTTCAACAGACCCCCTCTAGAA	GGGTGTCTCCCAGAAGCATCT
Human 18S	GTAACCCGTTGAACCCCATT	CCATCCAATCGGTAGTAGCG
Human ALDH1A1 promoter	AGGTCTACTTACCCAGCACTGAAAA	CCCTTTATCTACACCCTACCCTAAGTT
Human CD44 promoter	CTCCAGCCGGATTCAGAGAA	CCGAACCGTAAAACCTTGCA
Human Lgr5 promoter	CCTTGTGCGTCTGTTTAGGTCTCT	CCCACCCTTACCCCTTCATG
Mouse ALDH1A1	ATACTTGTCGGAT1TAGGAGGCT	GGGCCTATCTTCCAAATGAACA
Mouse ALDH3A1	CTCTAACCTGCGCAAGAATGAA	TCTGACGAGTCTTTGCCACAG
Mouse HPRT	GCAGTACAGCCCCAAAATGG	GGTCCTTTTCACCAGCAAGCT
Mouse Lgr5	CCTACTCGAAGACTTACCCAGT	GCATTGGGGTGAATGATAGCA
Mouse Nanog	CTCATCAATGCCTGCAGTTTTTCA	CTCCTCAGGGCCCTTGTCAGC
Mouse p53	CACGTACTCTCCTCCCCTCAAT	AACTGCACAGGGCACGTCTT
