# Supplementary material for: Mutant p53 gain of function underlies high expression levels of colorectal cancer stem cells markers
Source: Oncogene. Author manuscript; Available in PMC 2019 Apr 4. (PMC6448595; doi:10.1038/s41388-017-0060-8)
Supplement: Supplemental Material [file NIHMS1000581-supplement-Supplemental_Material.pdf]

Supplementary Figures:

Supplementary Figure 1

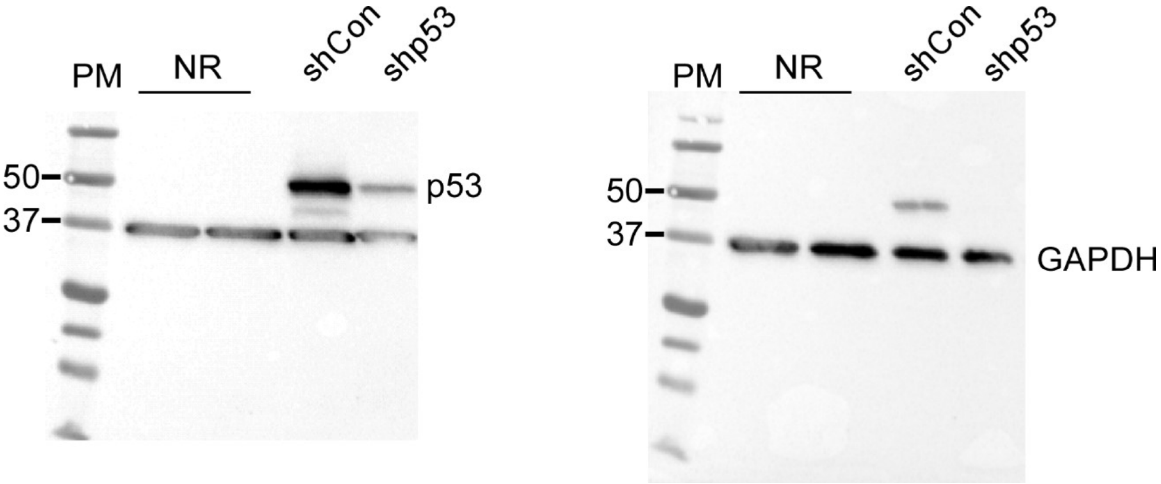

Supplementary Figure 1. Silencing mutant p53^{R273H,P309S} upon introduction of shRNA against p53. The colorectal adenocarcinoma derived cell-line, SW480 that endogenously expresses mutant p53^{R273H,P309S} was stably infected with shRNA against p53 (shp53) or with shRNA against non-specific sequence (shCon), as a control. The figure presents Western Blot displaying mutant p53 protein levels (left panel), and GAPDH (right panel) protein levels as loading control. This figure combines two independent detections of p53 and GAPDH of the same gel. NR; Non Relevant samples, PM; protein marker.

Supplementary Figure 2

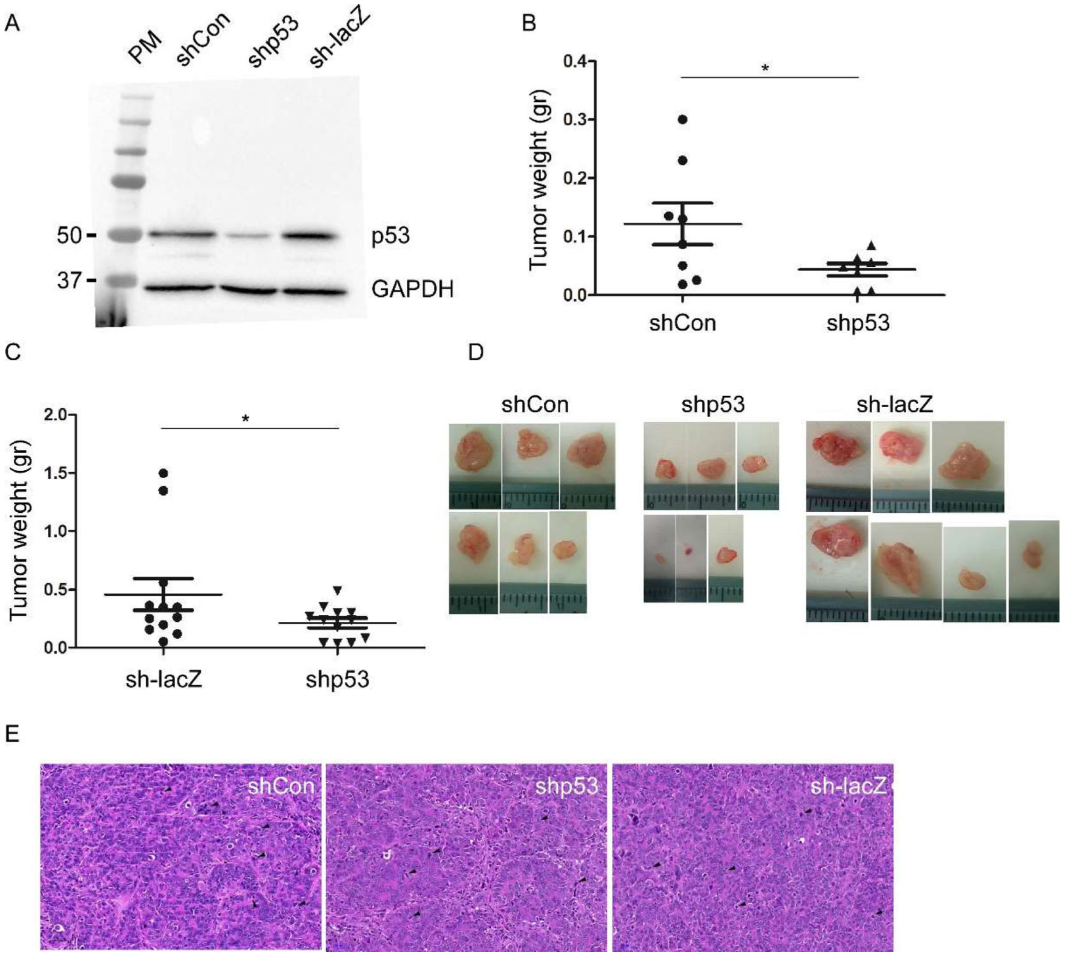

Supplementary Figure 2. Mutant p53 gain of function endows the colorectal cancer cell-line SW480 with higher oncogenic potential. The colorectal adenocarcinoma derived cell-line, SW480 that endogenously expresses mutant p53^{R273H,P309S} was stably infected with shRNA against p53 (shp53) to silence mutant p53, or with either shRNA against lacZ genes (sh-lacZ) or shRNA against non-specific sequence (shCon), as controls. (A) Western Blot displays mutant p53 protein levels. GAPDH serves as loading control. (B-C) The shCon and shp53 SW480 cell-lines (B) or sh-lacZ and shp53 cell lines(C) ($1X10^6$) were injected into nude mice, followed by tumor growth surveillance. After 20 days mice were sacrificed and tumors weight was measured. Asterisks denote statistical significance. (D) Representative photos of tumors presented in (B-C) that illustrate the significant differences in the size of tumors obtained from injection of the various SW480 cells. (E) Sections of the generated tumors were stained with hematoxylin and eosin (H&E) for pathological examination. Photos (magnification of X20) indicate on typical carcinoma with high mitotic rate. Some mitotic figures in each field are identified with arrowheads. PM; protein marker.

Supplementary Figure 3

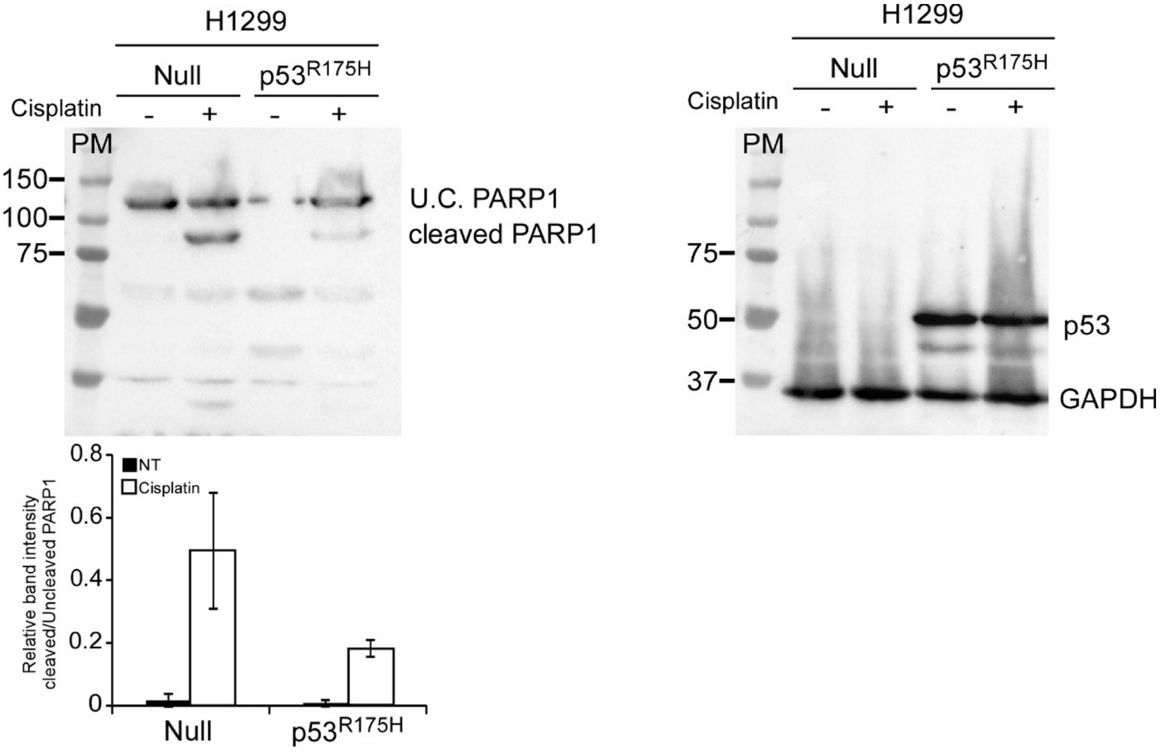

Supplementary Figure 3. Mutant p53 producing H1299 cells attenuate cisplatin induced cell death. Lung adenocarcinoma cell lines, H1299, that are null for p53 were stably infected with mutant $p53^{R175H}$. Cells were treated with cisplatin (2.5ug/ml) for 72 hours followed by cells harvesting and PARP-1 cleavage measurement by Western blot. Western Blot displaying both cleaved and uncleaved PARP-1 (left panel), and mutant p53 and GAPDH protein levels (right panel). GAPDH serves as loading control. This figure combines independent detections of PARP-1, p53 and GAPDH of the same gel. Quantification of un-cleaved PARP-1 bands is presented in the lower panels. U.C., uncleaved, PM; protein marker.

Supplementary Figure 4

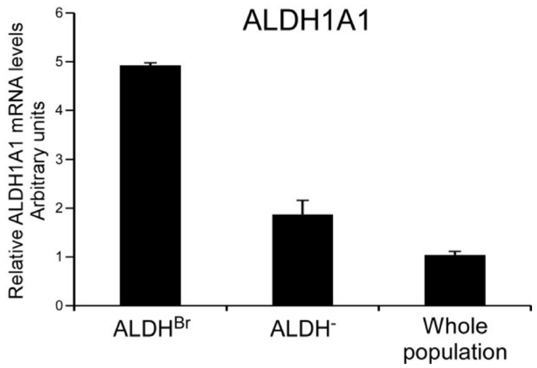

Supplementary Figure 4. ALDH1A1 is highly expressed in ALDH^{Br} SW480 sub-population. SW480 cells endogenously expressing mutant p53^{R273H,P309S} were sorted by FACS according to their ALDH activity levels. ALDH1A1 mRNA levels were measured by quantitative real-time PCR using specific primers.

Supplementary Figure 5

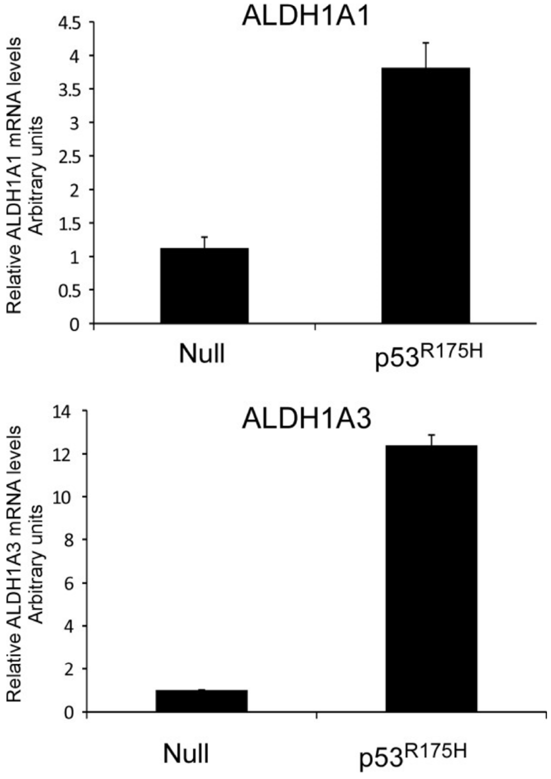

Supplementary Figure 5. ALDH1A1 and ALDH1A3 expression is higher in mutant p53^{R175H} expressing H1299 cells. Lung adenocarcinoma cell lines, H1299, that are null for p53 were stably infected with mutant p53^{R175H}. mRNA levels of ALDH1A1 and ALDH1A3 were measured by quantitative real-time PCR using specific primers.

Supplementary Figure 6

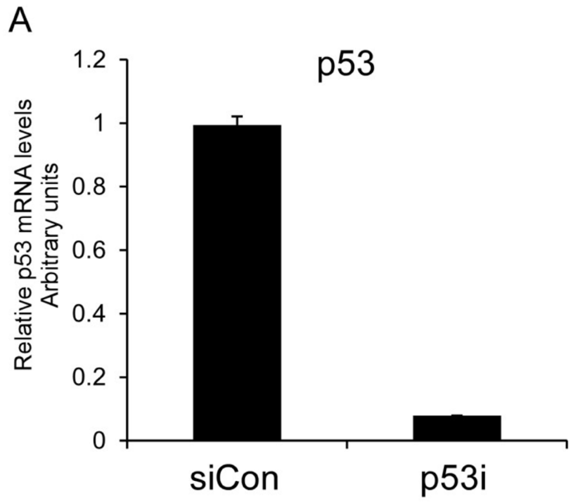

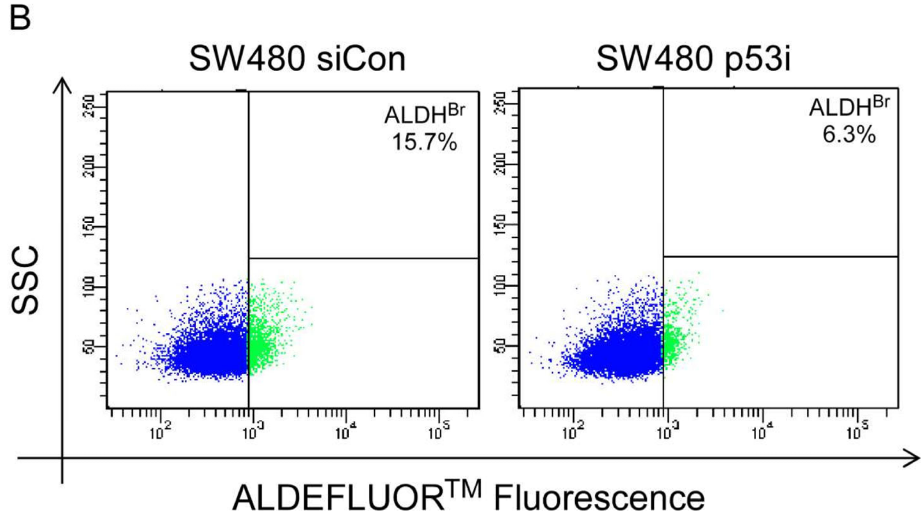

Supplementary Figure 6. Mutant p53^{R273H,P309S} increases the size of ALDH^{Br} sub-population within SW480 cellular population. SW480 expressing mutant p53^{R273H,P309S} were transiently transfected with siRNA against p53. 72 hours following transfection cells were subjected to ALDH activity assay. (A) mRNA expression levels of p53 as measured by quantitative real-time PCR using specific primers. (B) Distribution of cells sub-populations according to ALDH activity levels, as estimated by FACS.

Supplementary Figure 7

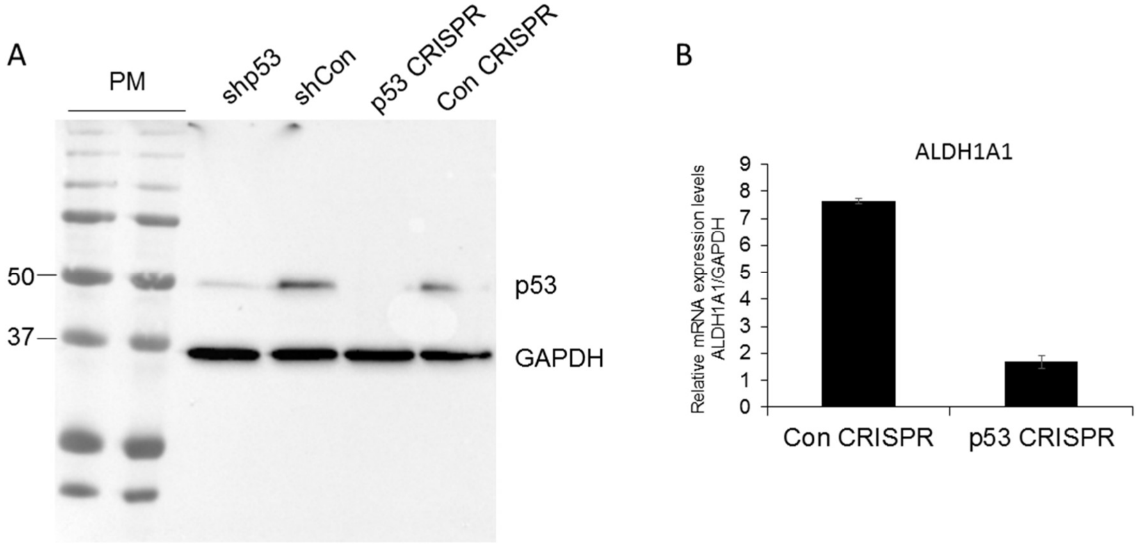

Supplementary Figure 7. ALDH1A1 mRNA levels are reduced upon knock-out of mutant p53^{R273H,P309S} genes using CRISPR/Cas9. SW480 expressing mutant p53^{R273H,P309S} were transiently transfected with pX330 p53 plasmid harboring gRNA against human p53 and Cas9. 48 hours after transfections single cells were plated on 96 well plates and upon clones expansion p53 levels were examined by western blot (A) and ALDH1A1 mRNA levels were examined by quantitative real-time PCR using specific primers (B). PM; protein marker.

Supplementary Figure 8

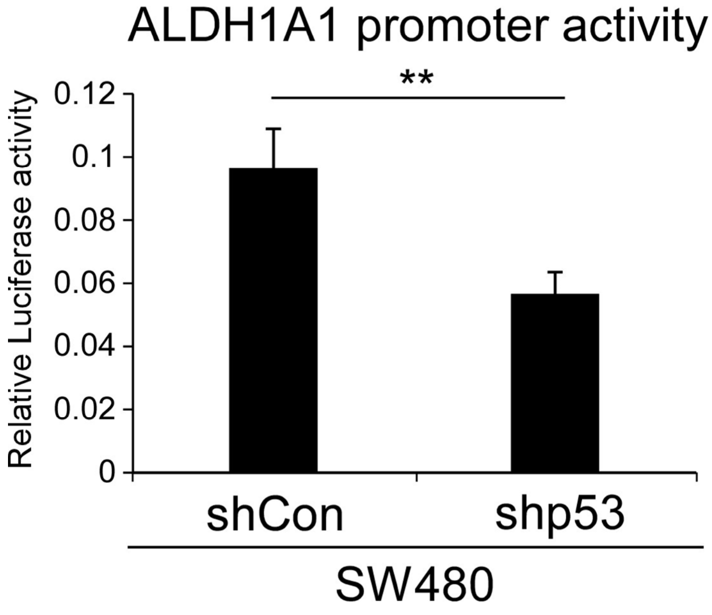

Supplementary Figure 8. Mutant p53^{R273H,P309S} induces ALDH1A1 promoter activity. SW480 cells were transiently co-transfected with Cypridina Luciferase reporter construct harboring ALDH1A1 1kb promoter sequence, Gaussia luciferase and CMV-GFP vector as controls for transfection efficiency. Luminescence values were estimated after 24h. In order to estimate transfection efficacy, values of the pCLuc-Basic2-ALDH1A1 were normalized to values of Gaussia luciferase.

Supplementary Figure 9

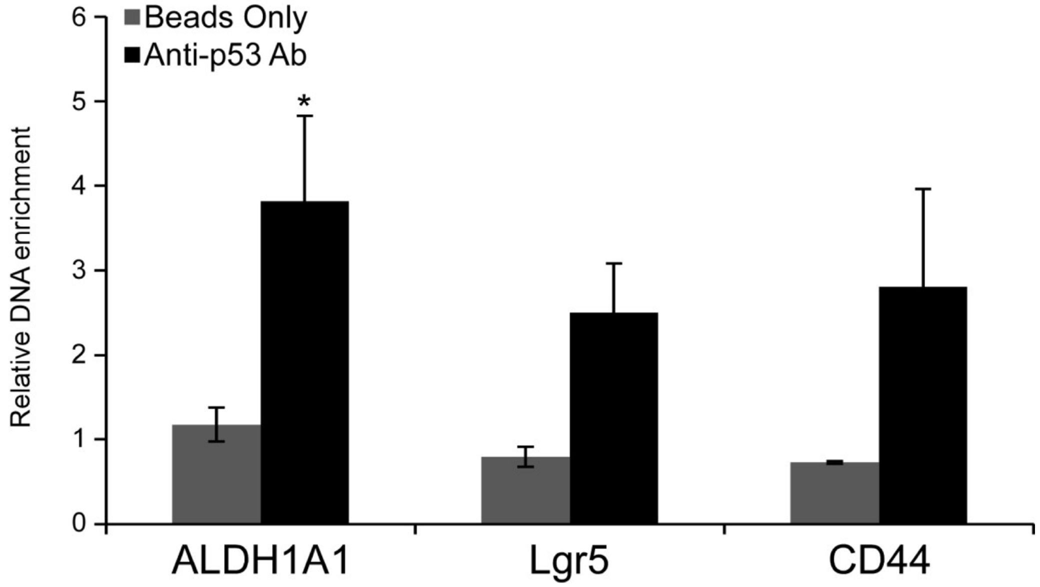

Supplementary Figure 9. Mutant p53^{R175H} binds to ALDH1A1, CD44 and Lgr5 promoters. ChIP analysis of SK-BR-3 cells. Endogenous mutant p53^{R175H} protein was immunoprecipitated using p53-specific antibody (anti-p53 Ab). Empty beads were used as a negative control (Beads only). qRT-PCR was performed using specific primers directed to ALDH1A1, CD44 and Lgr5 promoters. Values were normalized to 1% input. Results are average of three experiments. Error bars represent SE.

Supplementary Figure 10

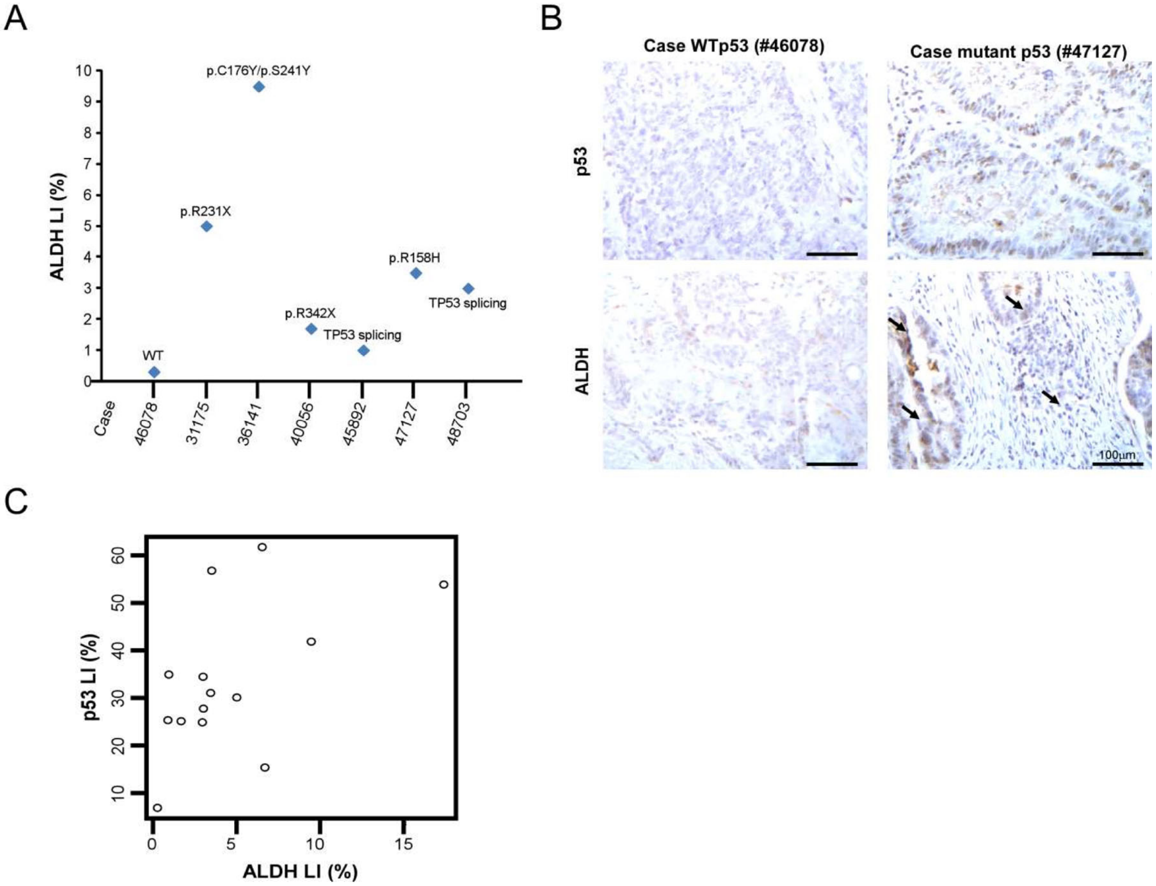

Supplementary Figure 10. Mutant p53 correlates with elevated ALDH levels in human colitis-associated CRC Tumor biopsies obtained from colitis associated colorectal carcinoma patients were immuno-stained for ALDH and p53. (A) Graph shows diagrammatic presentation of the status of ALDH along with the *TP53* sequencing data for all cases. (B) Representative photos of ALDH and p53 staining showing increased ALDH levels in a mutant $p53^{R158H}$ case compared to a WT one. Arrows denote positive ALDH staining. Scale bar: 100µm. (C) Tumor biopsies obtained from colitis associated colorectal carcinoma patients were immuno-stained for ALDH and p53. Scatter plot depicting the ALDH (%) and p53 (%) LI values for every single case. The Pearson correlation coefficient is 0.53.

Supplementary Figure 11

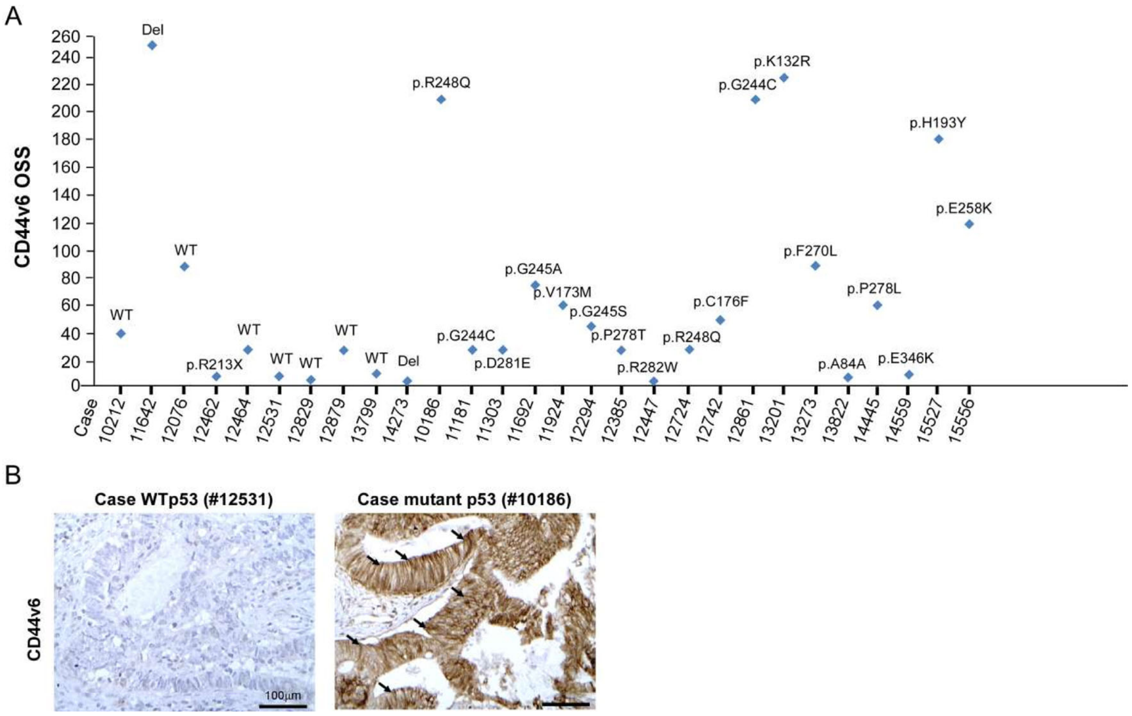

Supplementary Figure 11. Mutant p53 correlates with elevated CD44v6 in human CRC tumors. Tumor biopsies obtained from sporadic colorectal carcinoma patients were immuno-stained for CD44v6, and underwent *TP53* sequencing (A) Diagrammatic presentation of the status of CD44v6 along with the TP53 sequencing data for every individual case. OSS: Overall Staining Score. (B) Representative photos of CD44v6 staining depicting elevated CD44v6 levels in a mutant p53 case compared with a WTp53 one. Arrows denote positive CD44v6 staining. Scale bar: 100μm.

Supplementary Figure 12

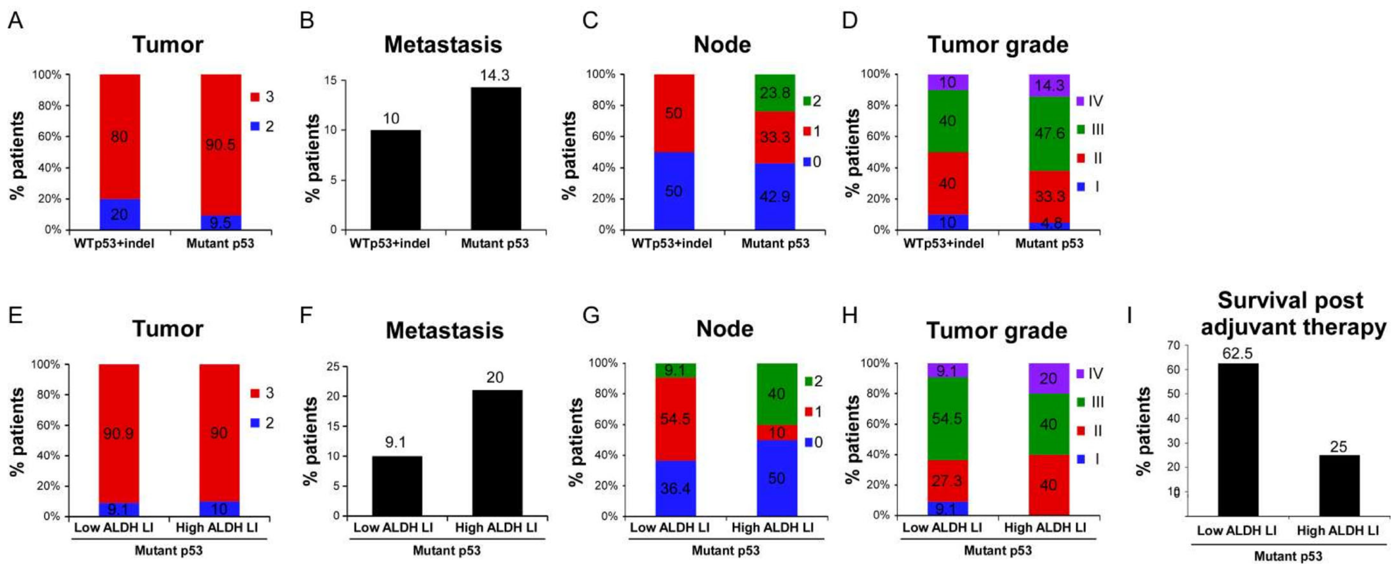

Supplementary Figure 12. **p53 missense mutations and high ALDHL1 levels are correlated with more aggressive human CRC tumors.** Primary tumor biopsies were obtained from the National Cancer Institute and the Mount Sinai School of Medicine where Patients' CRC staging was evaluated according to the TNM (Tumor, Node, Metastasis) staging system. (A-D) Comparative analysis of CRC staging parameters according to p53 status in tumors (i.e. WTp53/indel vs. p53 missense mutations). (E-H) Comparative analysis of CRC staging parameters of patients harboring tumors expressing p53 missense mutations, according to ALDH levels [i.e. high ALDH (ALDH LI>1.5 vs. low ALDH (ALDH LI<1.5)] in tumors. (I) Graph representing percentage of patients harboring tumors expressing p53 missense mutations that survived after adjuvant therapy in respect to ALDH levels detected in their tumors.

Supplementary Figure 13

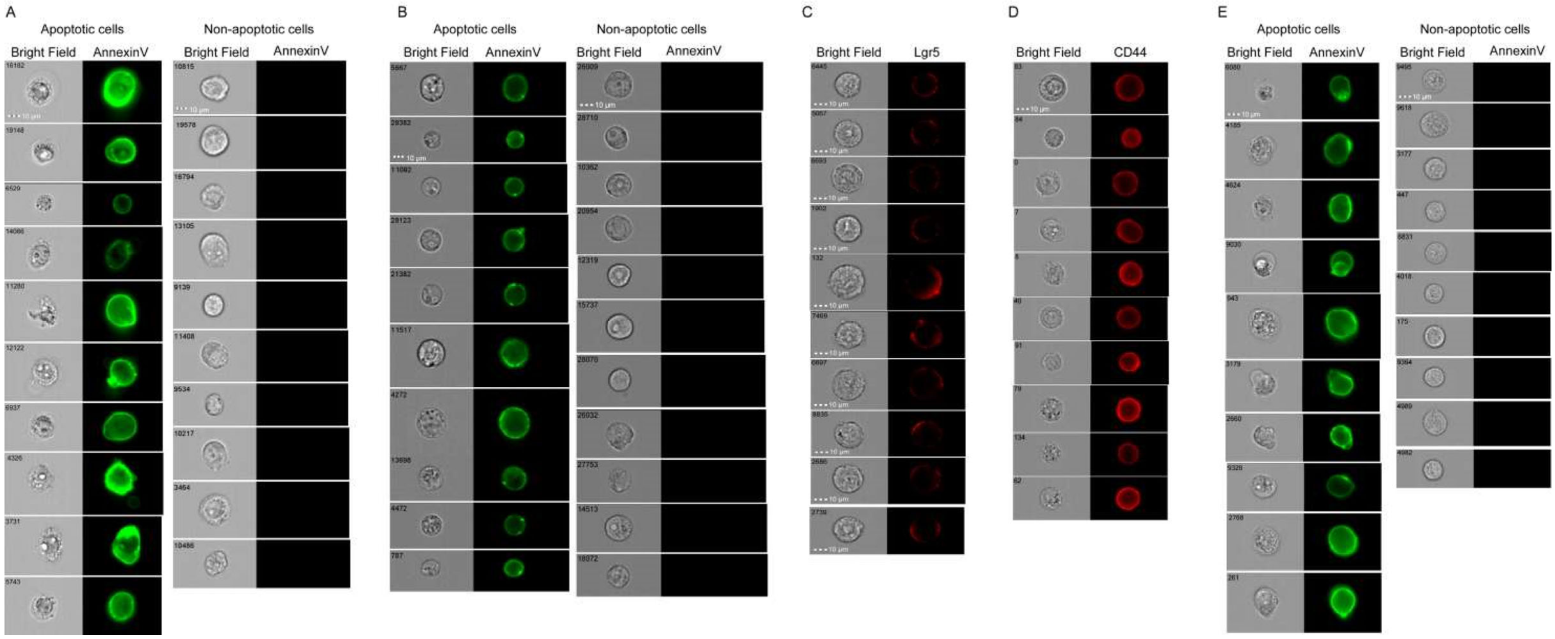

Supplementary Figure 13. ImageStream X photo gallery. Representative photos of cells analyzed by ImageStream X. (A) SW480 cells were treated with 5-FU and analyzed for apoptosis levels as indicated in Figure 1E. (B) RKO cells were treated with cisplatin and analyzed for apoptosis levels as indicated in Figure 1F. Photos are representative apoptotic (AnnexinV positive) cells vs. non apoptotic (AnnexinV negative) cells. (C) RKO cells were immunostained with anti-Lgr5 antibody and $Lgr5^{Br}$ sub-populations were assessed as described in Figure 2A-B. Photos are representative Lgr5 positive cells. (D) SW480 cells were immunostained with anti-CD44 antibody and $CD44^{Br}$ sub-populations were assessed as described in Figure 2E-G. Photos are representative CD44 positive cells. (E) SW480 cells were treated with cisplatin and analyzed for apoptosis levels as indicated in Figure 4C-D. Photos are representative apoptotic (AnnexinV positive) cells vs. non apoptotic (AnnexinV negative) cells.

Supplementary Table 1: Putative transcription factor binding sites in ALDH1A1, Lgr5 and CD44 genes, analyzed by Matinspector tool

ALDH1A1

Matrix Family	p-value	information
V$NKX1	0.011719	NK1 homeobox transcription factors
V$SORY	0.029025	SOX/SRY-sex/testis determining and related HMG box factors
V$AARF	0.02919	AARE binding factors
V$PAX1	0.02919	PAX-1 binding sites
V$ZF08	0.035866	C2H2 zinc finger transcription factors 8
V$HUB1	0.037953	HTLV-I U5 repressive element-binding protein 1
V$ETSF	0.042144	Human and murine ETS1 factors
V$PDX1	0.045319	Pancreatic and intestinal homeodomain transcription factor
V$DLXF	0.045793	Distal-less homeodomain transcription factors

Lgr5

Matrix Family	p-value	information
V$SIXF	0.008463	Sine oculis (SIX) homeodomain factors
V$GFI1	0.011916	Growth factor independence transcriptional repressor
V$PLZF	0.019487	C2H2 zinc finger protein PLZF
V$SORY	0.028097	SOX/SRY-sex/testis determining and related HMG box factors
V$PRDF	0.032766	Positive regulatory domain I binding factor
V$ARID	0.036085	AT rich interactive domain factor
V$HZIP	0.039627	Homeodomain-leucine zipper transcription factors
V$ETSF	0.040079	Human and murine ETS1 factors
V$MZF1	0.041178	Myeloid zinc finger 1 factors
V$ZF08	0.045162	C2H2 zinc finger transcription factors 8
V$BCDF	0.045604	Bicoid-like homeodomain transcription factors
V$PURA	0.047311	Pur-alpha binds both single-stranded and douple-stranded DNA in a sequence-specific manner
V$E4FF	0.049111	Ubiquitous GLI - Krueppel like zinc finger involved in cell cycle regulation

CD44

Matrix Family	p-value	Information
V$PAX7	0.000189	Paired box 7
V$RUSH	0.000541	SWI/SNF related nucleophosphoproteins with a RING finger DNA binding motif
V$YY1F	0.000683	Activator/repressor binding to transcription initiation site
V$ZF09	0.000851	C2H2 zinc finger transcription factors 9
V$HOXC	0.002174	HOX - PBX complexes
V$MYT1	0.002174	MYT1 C2HC zinc finger protein
V$DUXF	0.002479	Double homeobox factors
V$HDGF	0.003234	Hepatoma-derived growth factor
V$BRNF	0.003245	Brn POU domain factors
O$VTBP	0.003505	Vertebrate TATA binding protein factor
V$PIT1	0.003811	GHF-1 pituitary specific pou domain transcription factor
V$ABDB	0.005002	Abdominal-B type homeodomain transcription factors
V$HOXF	0.006788	Paralog hox genes 1-8 from the four hox clusters A, B, C, D
V$OCT1	0.006788	Octamer binding protein
V$PRDF	0.007616	Positive regulatory domain I binding factor
V$GFI1	0.009669	Growth factor independence transcriptional repressor
V$FAST	0.01045	FAST-1 SMAD interacting proteins
V$LTSM	0.010654	Localized tandem sequence motif
V$FKHD	0.017103	Fork head domain factors
V$DMRT	0.019115	DM domain-containing transcription factors
V$CHRF	0.019188	Cell cycle regulators: Cell cycle homology element
V$SORY	0.020296	SOX/SRY-sex/testis determining and related HMG box factors
V$HOMF	0.021232	Homeodomain transcription factors
V$CAAT	0.023606	CCAAT binding factors
V$LHXF	0.024864	Lim homeodomain factors
V$BZIP	0.026809	Heterodimers between bZIP family members
V$SIX3	0.028273	Sine oculis homeobox homolog 3
V$HMTB	0.031093	Human muscle-specific Mt binding site
V$BRN5	0.037866	Brn-5 POU domain factors
V$NKX1	0.038271	NK1 homeobox transcription factors
V$THAP	0.042195	THAP domain containing protein
V$TCFF	0.044133	TCF11 transcription factor
V$CART	0.049008	Cart-1 (cartilage homeoprotein 1)
